# Expiratory flow limitation during mechanical ventilation: real-time detection and physiological subtypes

**DOI:** 10.1186/s13054-024-04953-9

**Published:** 2024-05-21

**Authors:** Detajin Junhasavasdikul, Akarawut Kasemchaiyanun, Tanakorn Tassaneyasin, Tananchai Petnak, Frank Silva Bezerra, Ricard Mellado‐Artigas, Lu Chen, Yuda Sutherasan, Pongdhep Theerawit, Laurent Brochard, MAFAI VENT investigators

**Affiliations:** 1 Division of Pulmonary and Pulmonary Critical Care, Department of Medicine, Faculty of Medicine Ramathibodi Hospital, Mahidol University, 270 Rama 6 Rd. Rajthevi, Bangkok, Thailand; 2 Division of Critical Care, Department of Medicine, Faculty of Medicine Ramathibodi Hospital, Mahidol University, Bangkok, Thailand; 3 Keenan Research Centre, Li Ka Shing Knowledge Institute, St. Michael’s Hospital, Toronto, ON Canada; 4 Laboratory of Experimental Pathophysiology, Department of Biological Sciences and Center of Research in Biological Sciences, Federal University of Ouro Preto, Ouro Preto, Minas Gerais Brazil; 5 Interdepartmental Division of Critical Care Medicine, University of Toronto, Toronto, ON Canada; 6 Surgical Intensive Care Unit, Department of Anesthesia, Hospital Clinic, Barcelona, Spain; 7 Institute of Medical Science, University of Toronto, Toronto, ON Canada

**Keywords:** Mechanical ventilation, Positive-pressure respiration, Respiratory mechanics, Chronic airflow obstruction, Obesity

## Abstract

**Background:**

Tidal expiratory flow limitation (EFL_T_) complicates the delivery of mechanical ventilation but is only diagnosed by performing specific manoeuvres. Instantaneous analysis of expiratory resistance (Rex) can be an alternative way to detect EFL_T_ without changing ventilatory settings. This study aimed to determine the agreement of EFL_T_ detection by Rex analysis and the PEEP reduction manoeuvre using contingency table and agreement coefficient. The patterns of Rex were explored.

**Methods:**

Medical patients ≥ 15-year-old receiving mechanical ventilation underwent a PEEP reduction manoeuvre from 5 cmH_2_O to zero for EFL_T_ detection. Waveforms were recorded and analyzed off-line. The instantaneous Rex was calculated and was plotted against the volume axis, overlapped by the flow-volume loop for inspection. Lung mechanics, characteristics of the patients, and clinical outcomes were collected. The result of the Rex method was validated using a separate independent dataset.

**Results:**

339 patients initially enrolled and underwent a PEEP reduction. The prevalence of EFL_T_ was 16.5%. EFL_T_ patients had higher adjusted hospital mortality than non-EFL_T_ cases. The Rex method showed 20% prevalence of EFL_T_ and the result was 90.3% in agreement with PEEP reduction manoeuvre. In the validation dataset, the Rex method had resulted in 91.4% agreement. Three patterns of Rex were identified: no EFL_T_, early EFL_T_, associated with airway disease, and late EFL_T_, associated with non-airway diseases, including obesity. In early EFL_T_, external PEEP was less likely to eliminate EFL_T_.

**Conclusions:**

The Rex method shows an excellent agreement with the PEEP reduction manoeuvre and allows real-time detection of EFL_T_. Two subtypes of EFL_T_ are identified by Rex analysis.

*Trial registration*: Clinical trial registered with www.thaiclinicaltrials.org (TCTR20190318003). The registration date was on 18 March 2019, and the first subject enrollment was performed on 26 March 2019.

**Supplementary Information:**

The online version contains supplementary material available at 10.1186/s13054-024-04953-9.

## Take-home message

During mechanical ventilation, the rise of expiratory airway resistance (Rex) exceeding inspiratory resistance suggests tidal expiratory flow limitation (EFL_T_). The analysis of Rex can detect EFL_T_ in real-time and identify two physiological subtypes of EFL_T_, early or late in expiration.

**Supplementary Information:**

The online version contains supplementary material available at 10.1186/s13054-024-04953-9.

## Introduction

Expiratory flow limitation (EFL) is a phenomenon where the expiratory flow of the subject cannot increase despite a higher expiratory driving pressure at a given lung volume [1–3]. Particularly in patients with airway diseases, this can occur during tidal expiration and is referred to as “tidal EFL” (EFL_T_) [3]. The prevalence of EFL_T_ in the intubated patients seems to vary among populations, PEEP settings, and the detection technique used [3]. Up to one-third of patients on mechanical ventilation has been proven to have EFL_T_ in one study [4], emphasizing the frequentness of this condition. EFL_T_ leads to air-trapping and intrinsic PEEP, and has been described to be associated with several adverse clinical outcomes such as dyspneic sensation [5], asynchronies [6, 7], and extubation failure [8]. In many EFL_T_ patients, application of external PEEP does not significantly increase the total PEEP (PEEP_tot_) and plateau pressure (Pplat), a behaviour known as “PEEP absorber” [3, 9]. This leads to better ventilator triggering, less work of breathing, and improved ventilatory distribution of the tidal volume [10]. Contrarily, inability to recognize EFL_T_ might lead to the detrimental use of external PEEP in patients without EFL_T_ who lack the PEEP absorber behaviour, potentially causing hyperinflation and hemodynamic compromise [3, 9, 10].

The current methods for EFL_T_ detection rely on the measurements of expiratory flow during a specific manoeuvre, i.e., the intentional change of expiratory driving pressure, system resistance, or interruption of the flow [3]. On physiologic grounds, EFL_T_ was believed to be associated with dynamic airway collapse [1, 11–13]. Based on this, we hypothesized that real-time, instantaneously calculated expiratory airway resistance (Rex_i_) would show an immediate increase in Rex if EFL_T_ is present. Therefore, it could be an alternative way to detect EFL_T_ without the need to perform a manoeuvre.

The main objective of this study was to determine the agreement of EFL_T_ detection between the expiratory airway resistance (Rex) method and the PEEP reduction manoeuvre. We also intended to analyze the pattern of Rex curves in patients with EFL_T_ as an exploratory outcome.

## Methods

### Study design and setting

The Lung Mechanics, Asynchronies, and Flow Limitation in Assisted Invasive Mechanical Ventilation (MAFAI VENT) was a single-center prospective study that began in March 2019 at medical and respiratory ICUs of the Faculty of Medicine Ramathibodi Hospital, a university and tertiary-care hospital in Bangkok, Thailand. It was intended to explore the prevalence and demographic data of patients with EFL_T_. The study had been approved by Ramathibodi Hospital Committee for Research (MURA2018/1024) and was registered to the Thai Clinical Trials Registry (TCTR20190318003). Informed consent was obtained from the next of kin for each patient.

### Patients

Medical patients aged ≥ 15 years admitted to the medical intermediate ward and ICU due to acute respiratory failure requiring invasive mechanical ventilation were consecutively screened. All patients with ventilators capable of exporting data (Puritan-Bennett PB840/PB980 and Hamilton S1) were included. Patients with unstable oxygenation/hemodynamics were excluded (Additional file 1: Section S1, *Inclusion and exclusion criteria*).

Since the prevalence of EFL_T_ in our population was unknown at the start of the trial and potentially low (due to low incidence of obesity, modest average body size, young age), the sample size was calculated at the 7th month of recruitment suggesting that 334 patients would be required (Additional file 1: Section S2, *Sample size calculation*).

### Procedures

#### Patient preparation

With all patients in semi-recumbent position, baseline ventilator settings and waveforms were recorded for 1 min. Sedative/analgesic drugs, if any, were unchanged. The ventilator was then set to volume-controlled mode with 0.3 s end-inspiratory pause and the PEEP level set to 5 cmH_2_O. The tidal volume (Vt), peak inspiratory flow and respiratory rate (RR) were set to mimic the baseline of the patient. If the patient had shown significant spontaneous respiratory efforts, i.e., the actual RR more than the set RR, or the exhaled Vt (Vte) varied > 10% from a previous breath, a brief hyperventilation manoeuvre (a temporary increase of the set RR to 5 bpm above the actual RR) for 1–2 min was performed until no perceivable effort was achieved. The previous settings were then resumed and the stability of Vte ensured before proceeding to the next step.

#### PEEP reduction manoeuvre and lung mechanics measurements

While the waveforms were being continuously recorded and the ventilator in volume-controlled mode, PEEP level was abruptly reduced from 5 cmH_2_O to ZEEP (Additional file 1: Section S3, *standardized PEEP reduction manoeuvre*). At ZEEP, respiratory system mechanics were measured by 2 s end-inspiratory and end-expiratory pauses. PEEP was then returned to 5 cmH_2_O. The manoeuvre was repeated 3 times. All data were saved on a computer for off-line analysis.

### Determination of EFL_T_

#### EFL_T_ by PEEP reduction manoeuvre

**Fig. 1:**
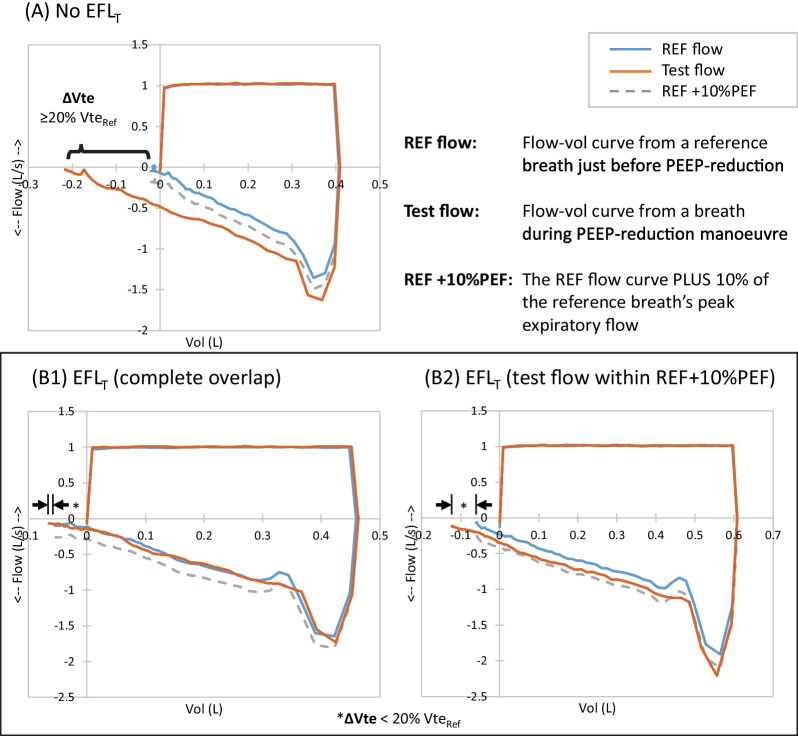
Flow–volume loops of representative patients undergoing PEEP reduction test from 5 cmH_2_O to ZEEP. In a sample patient without EFL_T_ (**Panel A**), the expiratory flow from the test breath will be higher (more negative) than and will deviate away from the reference breath, causing a large Vte change (ΔVte). In a patient with EFL_T_, the flow-volume curve of the test breath would overlap (**Panel B1**) or run very close and parallel (**Panel B2**) to the reference curve. The ΔVte is minimal (< 20% of Reference Vte). The flow-volume loops in panel B1 and B2 came from the same patient on the same occasion, but with a different set Vt

Our initial definition for EFL_T_ by PEEP reduction manoeuvre was the presence of substantial overlapping portion of the expiratory Flow-Volume curve of the test breath (with PEEP immediately reduced) and the curve of the preceding “reference” breath. After an early preliminary analysis, we observed variability in the flow of the test breath in the EFL_T_ patients. We then explored the existing set of data to find the best threshold regarding this phenomenon and formulated an operational definition for EFL_T_. EFL_T_ at ZEEP was present when the PEEP reduction manoeuvre from 5 cmH_2_O to ZEEP produced the following: (1) the Flow-Volume curve of the test breath had a significant portion (≥ 5% of the reference-breath Vte) running within an “envelope” curve. This envelope was defined as the reference-breath expiratory flow value plus a value of 10% of the reference-breath peak expiratory flow (PEF); and, (2) the increased exhaled tidal volume during the test breath was less than 20% of the reference breath (Fig. 1 and Additional file 1: Section S4).

Aided by a software (described below), 2 pulmonologists (D.J. and A.K.) independently interpreted the waveforms to determine EFL_T_. Any conflict of the results was resolved by a consensus.

#### EFL_T_ by Rex method

The Rex method was based on the perturbation method previously described in the literature [14–16]. Basically, the expiratory resistance at any instance (Rex_i_) can be calculated from the equation:

$$Rex_{i} = \frac{Instantaneous\, expiratory \,driving \,pressure}{{Instantaneous \,expiratory\, flow}}$$

The expiratory driving pressure is the effective alveolar pressure at that instance (Palv_i_) minus the pressure at the airway opening (Paw_i_).

$$Rex_{i} = \frac{{Palv_{i} - Paw_{i} }}{{\dot{V}_{i} }}$$

**Fig. 2:**
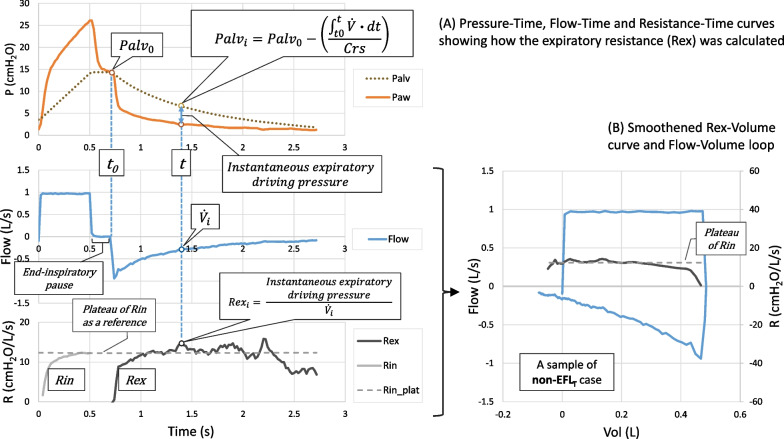
The calculation of the expiratory resistance (Rex) (**Panel A**). The Crs was pre-measured from a breath at the same PEEP level, with an end-expiratory pause to take the total PEEP into account. The pressure at the end-inspiratory pause is determined to be an “effective” alveolar pressure at time zero of expiration (Palv_0_). This was later calculated to an instantaneous alveolar pressure and expiratory resistance (Palv_i_ and Rex_i_). The inspiratory resistance (Rin) during a VCV breath with constant flow can be calculated by the same manner. The smoothened Rex curve and plateau of Rin were then plotted against the volume axis (**Panel B**). This was overlaid by the flow-volume loop of the same breath for better visualization of the point during expiration where the flow had changed. The Rex that significantly increases beyond the plateau of Rin should signify the presence of EFL_T_ at that particular PEEP level

At the beginning of expiration, with a short end-inspiratory pause (0.2–0.3 s), the effective Palv at time zero (Palv_0_) equals the measured Paw (Fig. 2, panel A). The Palv_i_ during the expiration then changes according to the amount of volume exhaled and the Crs. Thus, the equation can be re-written as:

$$Rex_{i} = \frac{{\left( {Palv_{0} - \left( {\frac{{\mathop \smallint \nolimits_{{t_{0} }}^{t} \dot{V} \cdot dt}}{Crs}} \right)} \right) - Paw_{i} }}{{\dot{V}_{i} }}$$

The inspiratory resistance (Rin) can also be calculated by the same approach. Using Palv_0_ as a starting point, and knowing the instantaneous inspiratory flow as well as the Crs, we back calculated into the inspiratory phase to find the Palv_i_, and eventually, the Rin_i_. In volume-controlled mode with constant flow, the Rin shows a plateau near the end of inspiration. We use this as a reference to determine the “expected” value of the Rex. Without EFL_T_, the value of Rex should be close to the plateau of Rin.

In order to better reflect the Rex change according to lung volume, we plotted the Rex on the volume axis instead of time. This was overlapped with the flow-volume loop of the same breath (Fig. 2, panel B). The breaths with end-expiratory pauses at ZEEP (for the purpose of Crs calculation) were selected for Rex calculation.

The following operational definition of EFL_T_ was used by the Rex criteria: a breath where the Rex_i_ value progressively increases along the expiration, to a value significantly higher (> 10 cmH_2_O/L/s) than the plateau of Rin. The total portion of this high resistance must be ≥ 5% of the Vte (Additional file 1: Section S5). Blinded to the result of the PEEP reduction manoeuvre, two pulmonologists (D.J. and A.K.) independently inspected the Rex curves at ZEEP to identify cases with EFL_T_.

### Data collection

We recorded the ventilator waveforms from the serial communication port (RS-232) to a computer. Off-line analyses were performed by our specialized software (MAFAI VENT waveform analyzer, Additional file 1: Fig. S1). The determination of EFL_T_ and lung mechanics were measured from the breaths with Vt stability (< 10% variation).

Baseline characteristics, ventilator settings and parameters were collected. Clinical outcomes were subsequently reviewed from the medical records.

### Statistical analysis and performance of the Rex method

The categorical data are presented by percentage. The continuous variables are presented by mean ± SD or median (interquartile range). Chi-Square or Fisher-Exact test was used for comparisons of categorical data based on the size of the samples. For continuous data, we used Student’s T-test for normally-distributed data and Mann–Whitney-U test for non-parametric data.

For the performance of EFL_T_ detection by the expiratory resistance (Rex) method, we compared its result with the PEEP reduction manoeuvre in a 2 × 2 contingency table. An independent validation was performed using an external dataset, the FLOWLY study (NCT03215316, Additional file 1: Section S6, *FLOWLY study*). Sensitivity, specificity, positive and negative predictive value, along with degree of agreement by Cohen’s kappa, are reported.

The data were analyzed by SPSS version 22.0 software (IBM SPSS Statistics, IBM corporation, New York, USA). A *p* value of < 0.05 was considered to be statistically significant.

## Results

In October 2020, we terminated the study with 339 analyzable cases. The flow diagram of the patients is shown in Additional file 1: Fig. S2.

### Prevalence and patient characteristics

The EFL_T_ prevalence was 16.5% by standardized PEEP reduction manoeuvre. The mean age was around 70, with a low prevalence of obesity (BMI ≥ 30 kg/m^2^) of 3.5%. The association between the baseline characteristics or clinical outcomes with EFL_T_ were shown in Table 1. The cases with EFL_T_ was found to have higher hospital mortality. The inspiratory airway resistance and the total PEEP were higher in EFL_T_ group, and the Crs is significantly lower (Additional file 1: Table S1).

**Table 1:** Baseline characteristics and clinical outcomes of patients with and without EFL_T_, as classified by the standardized PEEP reduction manoeuvre from PEEP 5 cmH_2_O to ZEEP

Parameters	All patients (n = 339)	Non-EFL_T_ group (n = 283)	EFL_T_ group (n = 56)	*P* value
Female, n (%)	163 (48.1)	130 (45.9)	33 (58.9)	0.075
Age, years	68.6 ± 17.5	68.0 ± 17.7	72.0 ± 16.5	0.119
Weight, kg	56.0 ± 13.9	55.0 ± 12.4	61.2 ± 18.8	0.02
Height, cm	159.4 ± 9.7	159.9 ± 9.7	157.3 ± 9.3	0.065
Body mass index, kg/m^2^	22.0 ± 4.9	21.5 ± 4.3	24.6 ± 6.5	0.001
Body mass index ≥ 30 kg/m^2^, n (%)	12 (3.5)	4 (1.4)	8 (14.3)	< 0.001
Glasgow Coma Score	12.5 ± 3.6	12.3 ± 3.7	13.7 ± 2.6	0.002
APACHE II score^a^	20.6 ± 7.2	20.3 ± 7.0	22.0 ± 7.9	0.13
SOFA score	4.0 (3.0–7.0)	4.0 (3.0–7.0)	4.0 (2.3–7.0)	0.579
PaO_2_/FiO_2_ ratio	343.9 ± 127.0	349.2 ± 127.1	317.3 ± 124.2	0.087
PaCO_2_, mmHg^b^	33.4 ± 7.4	32.9 ± 7.2	35.8 ± 7.8	0.007
*Main cause of intubation/mechanical ventilation, n (%)*				
Pulmonary cause	151 (44.5)	120 (42.4)	31 (55.4)	0.075
COPD with acute exacerbation	10 (2.9)	2 (0.7)	8 (14.3)	< 0.001
Acute asthmatic attack	6 (1.8)	4 (1.4)	2 (3.6)	0.259
Hemodynamic cause	64 (18.9)	49 (17.3)	15 (26.8)	0.098
Septic shock	53 (15.6)	38 (13.4)	15 (26.8)	0.012
Post cardiac arrest	11 (3.2)	11 (3.9)	0 (0)	0.222
Neurological cause	80 (23.6)	76 (26.9)	4 (7.1)	0.002
Post procedure/operation/airway protection	14 (4.1)	13 (4.6)	1 (1.8)	0.481
Cardiogenic pulmonary edema and volume overload	25 (7.4)	20 (7.1)	5 (8.9)	0.581
Other	5 (1.5)	5 (1.8)	0 (0)	0.595
*Underlying disease, n (%)*^*c*^				
Chronic cardiac disease	206 (60.8)	169 (59.7)	37 (66.1)	0.374
Chronic lung disease	76 (22.4)	55 (19.4)	21 (37.5)	< 0.001
COPD	30 (8.8)	20 (7.1)	10 (17.9)	0.001
Asthma	16 (4.7)	11 (3.9)	5 (8.9)	0.157
3-days accumulated I/O, L	+ 1.63 (+ 0.48 to + 3.28)	+ 1.65 (+ 0.49 to + 3.15)	+ 1.39 (+ 0.34 to + 3.41)	0.893
*Clinical outcomes*^*d*^				
ICU length of stay, day^e^	9.5 (5.6–16.1)	9.4 (5.6–16.1)	9.9 (5.5–18.9)	0.523
Hospital length of stay, day^f^	18.8 (11.6–32.6)	20.7 (11.7–33.0)	13.9 (9.7–31.6)	0.110
Hospital mortality; n (%)^g^	92 (28.1)	70 (25.8)	22 (39.3)	0.042

*APACHE II* acute physiology and chronic health evaluation II score, *COPD* chronic obstructive pulmonary disease, *SOFA* sequential organ failure assessment. Categorical variables are described as number (percentage); continuous variables are described as mean ± SD or median (interquartile range), as appropriate

^a^Data available in n = 274 (224 in non-EFL_T_ group and 50 in EFL_T_ group)

^b^Data available in n = 331 (276 in non-EFL_T_ group and 55 in EFL_T_ group)

^c^A patient could have multiple diseases

^d^Excluding cases intubated due to cardiac arrest

^e^n = 265 (223 in non-EFL_T_ group vs. 42 in EFL_T_ group), excluding patients who died in the ICU

^f^n = 220 (188 in non-EFL_T_ group vs. 32 in EFL_T_ group), excluding patients who died in the hospital or referred to other hospital

^g^n = 327 (271 in non-EFL_T_ group vs in EFL_T_ group 56), shown *p* value was adjusted for age (crude p-value = 0.043)

### Rex analysis

The Rex analysis at ZEEP revealed an EFL_T_ prevalence of 20.0%. The Rex method had 90.3% agreement when compared to the standardized PEEP reduction method (Table 2). In patients with EFL_T_ at ZEEP, EFL_T_ could be eliminated with the presence of external PEEP in 42.7% of cases (*P* < 0.001, Additional file 1: Table S2). In the validation dataset, the Rex method had resulted in 91.4% agreement with the PEEP reduction method (Additional file 1: Tables S3 and S4).

**Table 2:** The 2 × 2 contingency table for agreements between the Rex method and the standardized PEEP reduction method in the original dataset (MAFAI VENT)

	Rex analysis POSITIVE EFL_T_	Rex analysis NEGATIVE EFL_T_	Total n
PEEP reduction POSITIVE EFL_T_	45	11	56
PEEP reduction NEGATIVE EFL_T_	21	253	274
Total n	66	264	330

Using PEEP reduction from 5 cmH_2_O to ZEEP as a gold standard: The Rex analysis method provides 90.3% agreement (95% CI 86.6–93.3%), 80.4% sensitivity (95% CI 67.6–89.8%) and 92.3% specificity (95% CI 88.5–95.2%). The positive and negative predictive value of Rex were 68.2% (58.2–76.7%) and 95.8% (93.1–97.5%) respectively. The Cohen’s k is 0.68 (95% CI 0.58–0.78), i.e., substantial agreement [17]

### Re-analysis with a different pause duration

In Additional file 1: Fig. S3, we show that the measurements of Pplat and Crs using either a short (0.2–0.3 s) inspiratory pause or a longer one (2 s) were quite similar. The re-analysis of Rex for EFL_T_ detection based on Pplat using a 2 s pause also provided similar results to the original analysis using short pauses, and the agreements of EFL_T_ status between these two analyses were excellent with an agreement of 94.9% (Additional file 1: Fig. S3, Panel C and D).

### Exploratory analysis

#### Characteristics of Rex curves and proposed subtypes

Performing Rex analysis in a breath where the PEEP reduction manoeuvre took place gave us insights into the pathophysiology of EFL_T_ (Additional file 1: Fig. S4).

**Fig. 3:**
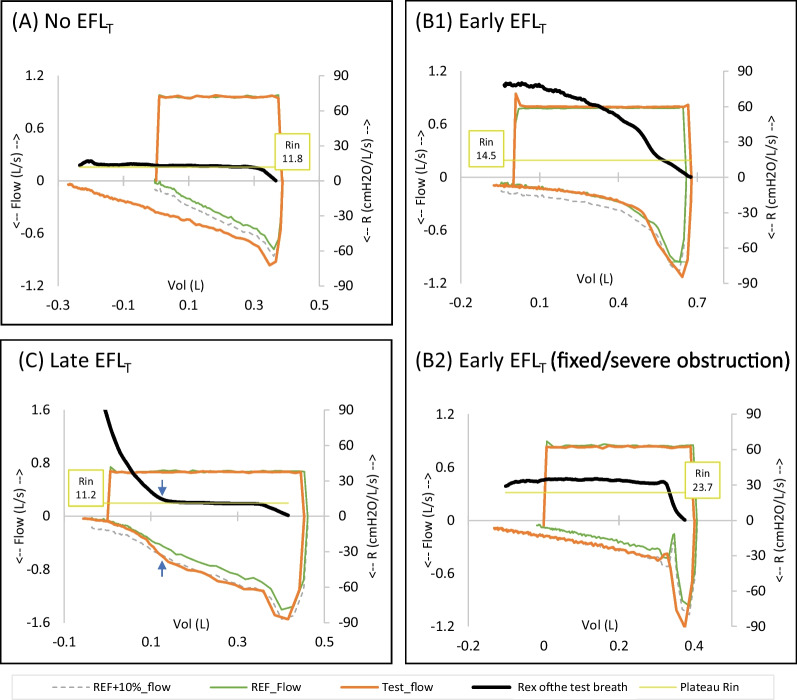
The three patterns of Rex curves (clockwise from the top left). **Panel A** The Rex pattern of patients without EFL_T_. Except for the initial part just before the “elbow” point, the Rex curve forms a constant line close to the value of Rin. **Panel B** The early EFL_T_, found mainly in patients with airway involvements. The Rex curves rise early from the beginning of the expiration, sometimes reaching a plateau. In *panel B1*, the Rex rises continuously beyond the value of Rin. In *panel B2*, i.e., severe early EFL_T_, the Rex sharply rises to a particular point and suddenly changes to a constant line which might be equal to or higher than the level of Rin. This probably indicates that the airway has reached its maximum possible resistance as it is mostly seen in patients with very severe airway obstruction or fixed lesion without dynamic change during inspiration and expiration. The flow-volume loop of a case with severe early EFL_T_ will usually show an initial flow spike, followed by a flatter low-flow part, i.e., the “dog-leg” pattern. The Rin is also generally higher than the other subtypes of EFL_T_. **Panel C** The late EFL_T_, found in patients with parenchymal/pleural/chest wall lesions. The Rex curve initially shows a constant phase equals to the Rin, similar to that seen in non-EFL_T_ cases. At a particular point, the Rex curve rises and deviates away from the Rin line. The blue arrows show the point where the Rex curve starts to deviate and the point where the flow-volume loop of the test breath begins to converge to the reference curve

We could classify the patients into 3 categories by determining where the resistance is rising in the Rex-volume curve (Fig. 3): no EFL_T_, Early EFL_T_, and Late EFL_T_. In early EFL_T_, the Rex curve continuously rises from the beginning of the expiration. On the contrary, in late EFL_T_, the Rex curve initially shows a constant phase equals to the Rin, before abruptly rising at the latter part of the exhalation. See Additional file 1: Section E, Fig. E1 for more examples.

These proposed subtypes of EFL_T_ also demonstrated different inspiratory resistance (Additional file 1: Fig. S5), suggesting different pathophysiological mechanisms.

#### Clinical characteristics of the subtypes

**Fig. 4:**
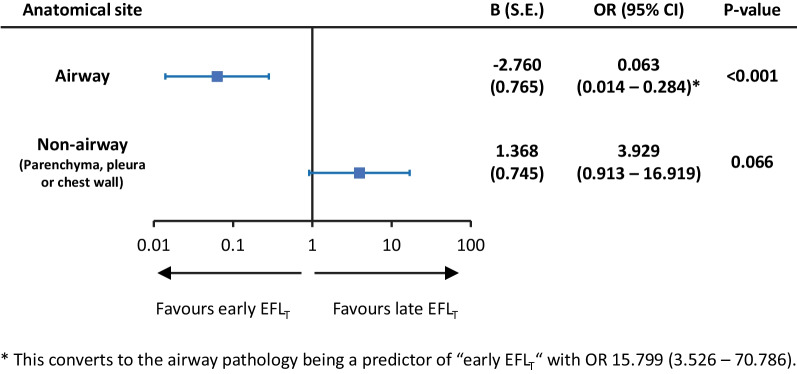
Binary logistic regression multivariable analysis showing anatomical site of pathology as independent predictors of “late EFL_T_”

Blinded to the EFL_T_ subtypes, two pulmonologists (D.J. and T.P.) independently reviewed the medical records and radiographs of EFL_T_ patients (closest to the time of waveforms collection) and verified whether the patient had active airway disease (e.g. COPD exacerbation, asthma attack, tracheobronchomalacia, endobronchial tumor) and/or parenchymal, pleural and chest wall abnormality (e.g. lung mass, pulmonary edema, pleural effusion, and obesity). The different subtypes of EFL_T_ were observed in patients with lesions involving different anatomical sites (Additional file 1: Table S5). We found that airway diseases significantly increased the likelihood of early EFL_T_, while patients with non-airway lesions, including obese patients, were more likely to have late EFL_T_ (Fig. 4).

## Discussion

Our study revealed the prevalence of EFL_T_ in mechanical ventilated patients to be from 16.5 to 20% according to the method used, a number in the low range compared to existing literature [4, 9, 18, 19]. This might be explained by the low prevalence of obesity, COPD and asthmatic exacerbation in our cohort, and also the exclusion of the most severe ARDS patients [20, 21]. The patients with neurological defect were less likely to have EFL_T_. These patients usually had their ETT in place due to airway-protection purpose, possibly explaining the lower prevalence of EFL_T_. The EFL_T_ group had higher adjusted hospital mortality [4], but other outcomes did not differ.

In this study, we have reached essential requirements when performing the PEEP reduction manoeuvre and developed a robust operational definition of EFL_T_. The initial PEEP should be standardized (i.e., 5 cmH_2_O) to avoid false-negative results. We found necessary to allow some variation in the flow of the test breath (up to + 10% of the reference PEF) when considering the overlapping of the Flow-Volume curve. The delta Vte between the test breath and the reference breath (< 20%) must also be considered. Remarkably, the sole criterion of the delta Vte has an almost-perfect agreement with the full criteria (Additional file 1: Section E, Table E1). Thus, the delta Vte of less than 20% when performing PEEP reduction from 5 cmH_2_O to ZEEP during the VCV mode might be a simple surrogate of EFL_T_, or screening tool, which is readily available at bedside everywhere.

We have introduced the use of Rex method. Once the Crs is known, the Rex method can be used to continuously monitor EFL_T_ without the need to perform any manoeuvre. A bench study in animals had also suggested a similar analysis and its capability to detect EFL_T_ which was provoked by application of negative pressure at the airway opening [22]. There might be a concern that the rising of Rex_i_ could be driven solely by the PEEP_i_ regardless of the EFL_T_ condition. Basically, Crs that derived from the plateau pressure minus the PEEP_i_ would be higher than the one using the plateau pressure minus the set PEEP. This would eventually result in a higher calculated Rex_i_. If this is the case, the Rex curve in non-EFL_T_ cases with the presence of PEEP_i_ (e.g. from too-short expiratory time) might also be rising, mimicking the EFL_T_ ones. In Additional file 1: Section E, Fig. E2, we have demonstrated that in a non-EFL_T_ patient with incomplete expiration, the Rex curve still forms a horizontal, constant line, not affected by the presence of PEEPi. The Rex method demonstrated a good agreement with the PEEP reduction manoeuvre, except for the fixed/severe obstruction that could be hard to detect by Rex method alone due to the Rex curve having a constant part, sometimes with the value closes to Rin (Panel B2 in Additional file 1: Section E, Fig. E1). Actually, 4 out of 11 cases with false-negative Rex result were caused by this subtype of EFL_T_. Some of the false positive results of Rex method could be favored by its higher sensitivity as compared to the PEEP reduction manoeuvre (Panel C in Additional file 1: Section E, Fig. E1). However, the clinical significance of these subtle EFL_T_ is debatable. The Rex analysis is applicable with data gathered from various model of ventilators and dedicated pneumotachometer. Moreover, although it was designed to be used with a VCV breath with an inspiratory pause, analysis of a PCV breath with a long inspiratory time enough to reach virtually zero flow at the end of inspiration is also possible. As for limitations, the Rex method needs precise numerical measurements and calculations by a software. Also, it assumes constant Crs along expiration which might not always be true, especially in cases with hyperinflation or inhomogeneous system with regional, small airway closure. It is worth mentioning that the Rex calculation includes the resistance of the ETT and the ventilator circuits. Severe occlusion at these sites could thus affect the Rex calculations. Furthermore, on physiological grounds, one might expect the Rex to be increasing as expiration proceeds even without the presence of EFL_T_ and also that Rex should be higher than the Rin due to lower lung volume during expiration [23]. This is not the finding of our study. It is possible that, in cases without hyperinflation, the change might be too subtle to be detected by our method, especially when the range of lung volume change is relatively small (a tidal volume that is well below the total lung capacity). Also, there were some studies reporting the Rin and Rex in healthy subjects with quiet breathing. The Rex was reported to be merely higher than the Rin at around 6–20% when measured by forced oscillation technique [24–26], or even only 4% in one study using interrupter technique [27]. As we had set the EFL_T_-defining threshold to be 10 cmH_2_O/L/s higher than the plateau of Rin, the physiological phenomenon of Rex being a bit higher than Rin would not affect the EFL_T_ identification. The difference of Rin and Rex was reported to be more prominent in adult patients with asthma, and even greater difference was found in patients with COPD [26], probably reflecting the effects of EFL_T_.

The Rex calculations require the estimation of Palv, which can be practically achieved by measuring Pplat. There are several possible time-points during an inspiratory pause at which Pplat could be measured, from an immediate moment just when the pause starts, to the point at several seconds later in longer pauses. The immediate pressure at the first point of zero flow after an inspiratory pause or “P1” had been suggested as a good surrogate for Palv [28, 29]. However, measurements of P1 require a specialized recording system with high frequency [29] and it would be unreliable if there was a phase-shift between the flow and the pressure data. For pauses with longer periods (e.g., 2 s or more), although they allow better equalization of pressure in cases with extreme lung inhomogeneity, they are not suitable for continuous monitoring. In this study, we had used Pplat after a short end-inspiratory pause of 0.2–0.3 s as a surrogate for Palv and for the Crs calculation. This duration was selected as it allowed standardization in the study and was pragmatic for real-time monitoring without introducing additional manoeuvre. We have demonstrated that the measurements of Pplat and calculated Crs from the short (0.2–0.3 s) inspiratory pauses and the long (2-s) pauses, and the re-analysis of Rex for EFL_T_ detection, were quite similar (Additional file 1: Fig. S3). However, precaution should be taken when using this short pause in cases with major pendelluft or stress relaxation.

**Fig. 5:**
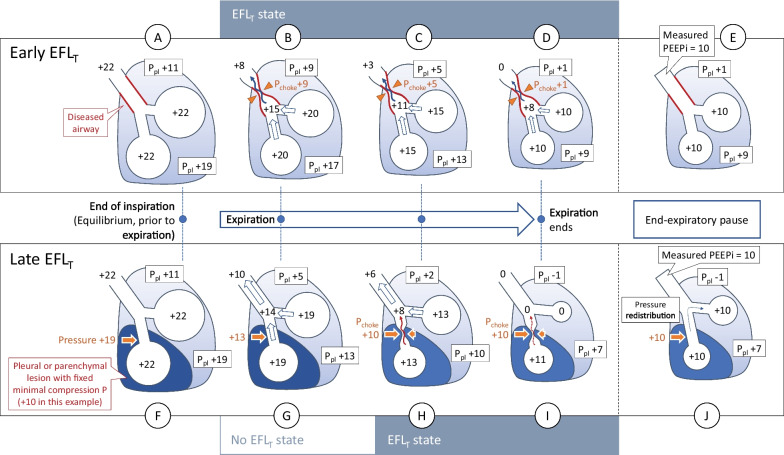
The proposed model to describe the pathophysiology of the two subtypes of EFL_T_. In the early EFL_T_ (**Panel A–E**), the main pathology involves the airway and might be enhanced by low lung elastance. This generates EFL_T_ with varying choke pressure (P_choke_) according to the pleural pressure (P_pl_) at that moment. The EFL_T_ develops early and persists along the expiration (**Panel B–D**). In the late EFL_T_ (**Panel F–J**), the main pathology is a lesion exerting a certain fixed minimal pressure on some part of the lungs (usually the dependent part). During the early phase of expiration, the pressure inside the lumen of the airway traversing the lesion is still higher than the compressing force by the lesion and thus the EFL_T_ is not present at this point (**Panel G**). The intraluminal, pleural, and the compressive pressure of the lesion decrease along expiration, but at some point, the compressive pressure of the lesion reaches its threshold and will not go further down (fixed at + 10 cmH_2_O in this example) while the pressure in the lumen continues to lower. When the intraluminal pressure equals the compressive pressure, the choke point and EFL_T_ occurs (**Panel H**). This segment of trapped air with low expiratory flow affects the global Rex_i_ calculation, hence the start of Rex_i_-rise. This P_choke_ is held constant along the rest of expiration and finally becomes the one that determines the PEEPi (**Panel J,** 10 cmH_2_O)

We observed two subtypes of EFL_T_ that have not been, to our knowledge, described before. One bench study recognizing the dynamic of Rex only described a single pattern, compatible with the late EFL_T_ [22]. Even without the Rex analysis, the early and late EFL_T_ subtypes can already be distinguished by the PEEP reduction manoeuvre which would show different overlap pattern between the flow-volume curves. Although it is arguable that the two subtypes might actually be the two extremes of one continuum process that occurs at different phase of expiration depending on its severity, our findings suggest that the early EFL_T_, with complete overlap of the whole flow-volume curves and previously described as “total” EFL_T_ [2], is not simply a more severe version of the late EFL_T_ (with partial overlap of flow-volume curves, “partial” EFL_T_), and vice versa. It is likely that the two subtypes originated from different mechanisms. The inspiratory resistance was different and the dominant sites of pathology were not the same in the two subtypes. Also, in early EFL_T_, application of external PEEP to eliminate EFL_T_ seems to be less effective (Additional file 1: Section E, Table E2) and probably poses more risk of hyperinflation. We have proposed a model to describe the different pathophysiology behind the two subtypes of EFL_T_ (Fig. 5). This new concept still needs to be confirmed. It should be noted that the Palv and the Rex calculated by our method are based on a hypothesis as described above and a one-compartment lung model. This might not reflect the actual or regional value in real patients.

The physiology of the intrinsic PEEP and EFL_T_ have been studied for a long time [3, 10]. In 1988, Smith and Marini had described the benefit of external PEEP application in mechanically-ventilated patients with chronic airflow obstruction [16]. Rex was found to be reducible when external PEEP was increased. The work of breathing was greatly decreased in patients whose peak pressures remained stable after PEEP increment [16]. This supported the concept that PEEP application would be beneficial only in EFL_T_ patients [3, 10]. Later, it was found that the response to PEEP among EFL_T_ patients could be different. When external PEEP was applied, the PEEP_tot_ was stable only in about half of EFL_T_ patients while the other half developed hyperinflation [9]. Our proposed subtypes of EFL_T_ might be able to explain this finding. Patients with late EFL_T_ subtype seem to be in favour of PEEP application (having lower PEEPi and greater chance of EFL_T_ being eliminated) while patients with early EFL_T_ might be more likely to develop hyperinflation (Additional file 1: Section E, Table E2). PEEP absorber behaviour in different subtypes of EFL_T_ is a subject for future research.

Our study is the largest cohort studying EFL_T_ to date with 339 patients in the primary analysis (and 440 cases analyzable by Rex method). Waveforms were analyzed in digital format and were precisely calculated by a dedicated software. The classification of EFL_T_ and their subtypes were reviewed by 2 independent pulmonologists. Although the rise of Rex value and its potential to be used as a tool for EFL_T_ detection had been suggested in some previous reports [16, 22], this is the first time it is used in a large clinical cohort, without additional manoeuvre, compared to the standard method (the PEEP reduction manoeuvre), and validated by an independent dataset from another center. Nevertheless, our study is not without limitations. We had tried to develop robust definitional criteria for EFL_T_, both for the PEEP reduction manoeuvre and the Rex analysis, but these criteria can still be considered as questionable. As the sedation is not mandatory, some subjects still exerted spontaneous efforts which might interfere with the data interpretation. We overcame this by briefly hyperventilating the patients who had excessive drive and performing all manoeuvres for 3 times, excluding the ones with significant variation in breathing pattern. The hyperventilation session, however, could affect lung mechanics and the partial pressure in the alveolar gas which might eventually alter the baseline EFL_T_ status. We did not include the most severe ARDS patients with high risk of hypoxemia and the phenomenon of airway closure has not been explored. Regarding Rex analysis, we had assumed a simple linear one-compartment model of the respiratory system for Rex calculation. When the lungs are in the state of extreme inhomogeneity, the calculated Rex might not reflect the actual resistance and caution is required for the interpretation of the absolute value measured. The concept of interpreting Rex as indicating EFL_T_ does not rely on its numerical value, but rather a pattern that deviates from the non-EFL_T_ one. The comparison of the Rex value to quantitatively determine the EFL_T_ severity between different patients would still require further confirmation study. Furthermore, Rex can easily be disturbed by the presence of spontaneous efforts or leakages, and would require a specialized software to calculate. We used the raw data exported from the ventilators that lack the compensation for tubing resistance and compliance. Still, we believe this had probably little effect on Rex calculation, as the agreement with the PEEP reduction manoeuvre was still good. Moreover, in ventilators with built-in sensors distant from the wye-piece, the “phase-shift” between the flow and pressure signal can occur (Additional file 1: Section E, Fig. E1, see *“Remark”*). Notwithstanding this point, since the definition of the EFL_T_ by the Rex method was defined as a “portion” of Rex that was higher than the threshold at any lung volume (no matter if it was earlier or later on), the phase-shifting of the Rex curve would not have a significant effect for EFL_T_ interpretation. Regarding the pathogenesis, the determination of the anatomical sites involved in EFL_T_ patients is subjective and when there is more than one site involved, it is unpredictable which one would play a dominant role. Also, the association between the non-airway lesions and late EFL_T_ has not reached statistical significance. The measurements of pleural pressure had not been performed, thus the specific relationship between the pleural pressure and the subtypes of EFL_T_ cannot be established.

## Conclusion

The prevalence of EFL_T_ differs among population and the method being used for detection. The presence of EFL_T_ is associated with more complicated clinical outcome and these warrant active detection of EFL_T_ in everyday practice. Simple measurements of Vte difference during a PEEP reduction manoeuvre may be a practical way of screening for EFL_T_. The analysis of the expiratory resistance (Rex) is an alternative method to detect and classify the EFL_T_ that does not require a manoeuvre. Finally, subtypes of EFL_T_ according to different pathophysiology can be observed. The different response to PEEP application among these two subtypes might affect the clinical decision to use PEEP.

### Supplementary Information

Supplementary Material.

## Data Availability

The datasets used and/or analysed during the current study are available from the corresponding author on reasonable request.

## Abbreviations

ARDS: Acute respiratory distress syndrome
COPD: Chronic obstructive pulmonary disease
Crs: Respiratory system compliance
EFL: Expiratory flow limitation
EFL_T_: Tidal EFL
ICU: Intensive care unit
Palv: Alveolar pressure
Paw: Pressure at the airway opening
PCV: Pressure-controlled ventilation
PEEP: Positive end-expiratory pressure
PEEP_i_: Intrinsic PEEP
PEEP_tot_: Total PEEP
PEF: Peak expiratory flow
Pplat: Plateau pressure
Rin: Inspiratory airway resistance
Rex: Expiratory airway resistance
Rex_i_: Instantaneous expiratory airway resistance
RR: Respiratory rate
VCV: Volume-controlled ventilation
Vt: Tidal volume
Vte: Exhaled tidal volume
ZEEP: Zero end-expiratory pressure
