# Supplementary material for: Expiratory flow limitation during mechanical ventilation: real-time detection and physiological subtypes
Source: Crit Care. 2024 May 21;28:171. doi: 10.1186/s13054-024-04953-9 (PMC11106966; doi:10.1186/s13054-024-04953-9)
Supplement: Supplementary file 1 — Supplementary Material. [file 13054_2024_4953_MOESM1_ESM.pdf]

Supplemental documents

Expiratory flow limitation during mechanical ventilation: real-time detection and physiological subtypes

Detajin Junhasavasdikul, Akarawut Kasemchaiyanun, Tanakorn Tassaneyasin, Tananchai Petnak, Frank Silva Bezerra, Ricard Mellado-Artigas, Lu Chen, Yuda Sutherasan, Pongdhep Theerawit, and Laurent Brochard; for the MAFAI VENT investigators.

Online Data Supplement

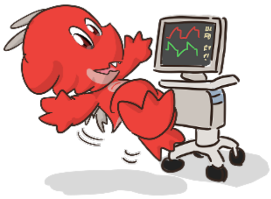

The Lung Mechanics, Asynchronies, and Flow Limitation in Assisted Invasive Mechanical Ventilation (MAFAI VENT) study.

Detajin Junhasavasdikul, MD. et, al.
Faculty of Medicine Ramathibodi Hospital. Mahidol University. Bangkok, Thailand.

Table of contents

Section S: Supplemental documentation and the findings of the trial 3

Section S1: Inclusion and exclusion criteria 4

Section S2: Sample size calculation 5

Section S3: Standardized PEEP reduction manoeuvre 6

Section S4: Development of EFL_T criteria for the PEEP reduction manoeuvre 7

Section S5: Development of EFL_T criteria for the Rex method 10

Section S6: FLOWLY study (validation set for Rex analysis) 12

Table S1: Baseline ventilator settings, parameters, and lung mechanics at baseline 13

Table S2: The presence of EFL_T at ZEEP and at PEEP level ≥ 5 cmH_2O 14

Table S3: The agreements between the Rex method and the PEEP reduction (FLOWLY study) 15

Table S4: Characteristics of patients in the validation set for Rex analysis (FLOWLY study) 16

Table S5: The distribution of the anatomical site of pathology between each subtype of EFL_T 17

Figure S1: Waveform data analyses in more details 18

Figure S2: The flow diagram of the patients 19

Figure S3: Differences of lung mechanics and Rex agreements from short and long pauses 20

Figure S4: Rex calculations in different breaths in a patient with EFL_T 21

Figure S5: The differences of inspiratory resistance between subtypes of EFL_T 22

Section E: Examples of cases and additional materials for discussion 23

Table E1: The agreements between the "delta Vte only" VS the "full criteria" 24

Table E2: Response to external PEEP application among patients with 2 subtypes of EFL_T 25

Figure E1: More examples of Rex curves 26

Figure E2: PEEPi in non-EFL_T cases does not affect the Rex curve 27

References for supplemental documents 28

The MAFAI VENT investigators 29

Section S

Supplemental documentation and the findings of the trial

Section S1: Inclusion and exclusion criteria

Inclusion criteria

1) Using the ventilators those were capable of exporting pressure-time and flow-time signal via a serial communication port (i.e., Puritan-Bennett PB840/PB980 and Hamilton S1, which comprised around 80% of the ventilators being used in our center)

2) The informed-consent could be obtained and EFL_T detection manoeuvres began within 48 hours after the ventilator connection

3) Permission from the attending doctor to go through the study manoeuvre was granted

Exclusion criteria

1) Uncorrectable hypoxemia (SpO_2 <90%)

2) Uncorrectable hemodynamic instability (i.e., mABP <60 mmHg, systolic arterial pressure >180 mmHg, heart rate <40 or >160/min)

3) Had already been enrolled in this study and was re-intubated within 48 hours from the previous enrollment

4) Denied to participate

5) Severe ARDS patients with high risk of severe hypoxemia at ZEEP (judging by clinicians)

Section S2: Sample size calculation

One of the well-known factors associated with EFL_T in previously reported studies is obesity (BMI ≥ 30 kg/m^2) [Ref E1, E2, E3, E4, E5]. However, the prevalence of obesity in Asian population is much lower than those observed in western countries. Since there was no study to depict the prevalence of EFL_T in our population, we initially aimed to perform the study for 1 year. After 7 months of recruitment and 217 analyzable cases had been collected, we performed an interim data analysis. The prevalence of EFL_T by the PEEP reduction method was about 13% (11.3% in non-obese and 38.5% in obese patients). The preliminary multivariable analysis had determined that the previously-described factor, BMI ≥ 30 kg/m^2, had the odds ratio of 2.74 (95%CI 0.67-11.22) to predict the EFL_T (p= 0.161). This was caused by the low prevalence of obese patients (13 out of 217 cases, i.e. 6.0%). We thus calculated **the sample size needed to determine the effect of obesity to predict the presence of EFL_T** using the following formula [Ref E6]:

$$n = (A + B)^2/C$$

Where $A = Z_\alpha\sqrt{\mathrm{P}(1-\mathrm{P})(1/q_1 + 1/q_0)}$

$$B = Z_\beta\sqrt{P_1(1-P_1)(1/q_1) + P_0(1-P_0)(1/q_0)}$$

$$C = (P_1 - P_0)^2$$

We aimed for 20% power (β = 0.2, Z_β = 0.84), with 95% confidence interval (α = 0.05, Z_α = 1.96). The parameters q_0 and q_1 are the proportion of non-obese and obese patients, respectively (q_0 = 0.94, q_1 = 0.06). P_0 and P_1 are prevalence of EFL_T in the two groups (0.113 and 0.385, respectively), where P = pooled proportion = 0.13. For the continuity correction [Ref E7], the additional number needed in the non-obese group = $1/(q_1 \times |P_1 - P_0|)$ = 61 cases, and the additional number needed in the obese group = 4 cases. **From these calculations, the sample size required for our study was 334 cases.** The extension of the study has been approved by Ramathibodi hospital's Committee for Research.

Section S3: Standardized PEEP reduction manoeuvre

Among different methods for EFL_T detection, e.g., negative expiratory pressure, manual abdominal compression, interrupter technique; we had chosen the PEEP reduction manoeuvre as a reference test. The PEEP reduction manoeuvre requires no specialized apparatus and it is independent from the operators [Ref E4], making this the most suitable method for a clinical study. One large clinical cohort study had also implemented this manoeuvre for studying EFL_T, supporting the feasibility of the method [Ref E5].

In previous literatures using PEEP reduction manoeuvre, there was no consensus regarding the level of initial PEEP level to be used in the manoeuvre. Also, the level of PEEP change (difference between the initial PEEP and the test PEEP) seems to be varied, and the value of 2, 3, or 6 cmH_2O has been reported for EFL_T detection [Ref E4, E5, E8, E9, E10].

During our pilot analysis with PEEP reduction manoeuvre from various initial PEEP levels (between 5 - 10 cmH_2O) to ZEEP, there were **conflicting results when different set-PEEP were used in the same patient**. In some patients with clinically suspected EFL_T (e.g., substantial PEEPi with PEEP absorption behavior), PEEP reduction from the level that was greater than the measured PEEPi showed negative results. In these same patients, repeated test with an initial set-PEEP level lower than or close to the PEEPi yielded positive results. **The findings suggested that using too high initial set-PEEP could yield a false-negative result, as the manoeuvre might not be able to detect EFL_T that occurs below the level of an initial set-PEEP**. Starting from a higher set PEEP would make the PEEP reduction test less sensitive due to a larger part of the expiration that is not flow limited. This can also be demonstrated by the concept of the PEEP absorption behavior [Ref E11] and hyperinflation.

Considering the level of PEEPi ≥ 5 cmH_2O to be clinically significant [Ref E11], and aiming to detect the presence of EFL_T at ZEEP, we had decided to utilize the **"standardized PEEP reduction manoeuvre"** i.e., PEEP reduction from 5 cmH_2O to ZEEP. Using the initial PEEP of 5 cmH_2O was also practical, since it was the level of "routine" set PEEP in patients who had no indication for a specific PEEP level in our center.

Section S4:
Development of EFL_T criteria for the PEEP reduction manoeuvre

In existing studies using PEEP reduction, EFL_T was determined mainly by visual overlap of the Flow-Volume curves between the test breath and the reference breath [Ref E5, E8, E9, E12]. The numerical value which defines “overlapping” of the curves existed only in some small studies using PEEP reduction and negative expiratory pressure (NEP) method with <5% increment of flow in the test breath (as compared to the reference) defined as EFL_T [Ref E10, E13, E14]. This cut-off value in NEP studies was originated from a pilot study in infants [Ref E13]. This requires the development of new substantial criteria defining EFL_T in an adult study deploying PEEP reduction manoeuvre.

In a small study (10 patients) using PEEP reduction method, the PEEP level was reduced only by 2 cmH_2O from the reference breath and EFL_T was considered to exist if the test flow curve overlapped with the reference flow curve for >10% of the volume slice [Ref E10]. In our study which utilized PEEP reduction of 5 cmH_2O, the flow was expected to be increased at a larger extent and the portion that overlaps with the reference was expected to be less. We then decided to define EFL_T with lower threshold of overlapping, i.e., 5% instead of 10% Vte.

With pilot data collection, we have found that within the same EFL_T patient underwent PEEP reduction, the Flow-Volume curve of the test breath might completely overlap with the reference, or it might be a little bit higher, running close and parallel to that of the reference breath **(Figure 1, main text)**. In order to allow the flow variation in EFL_T cases, we calculated the 10% of the reference breath’s peak expiratory flow (PEF). This derived value would then be added to the reference flow to generate an envelope of reference flow + 10% PEF. This gave the preliminary EFL_T criterion: a test breath showing an expiratory Flow-Volume curve with ≥ 5% of Vte running within the threshold of REF curve + 10%PEF (i.e., within the envelope of the dashed line).

However, there were a number of non-EFL_T cases with subtle increase of the expiratory flow during PEEP reduction, which might be misclassified as EFL_T cases. In this case, a significant portion of

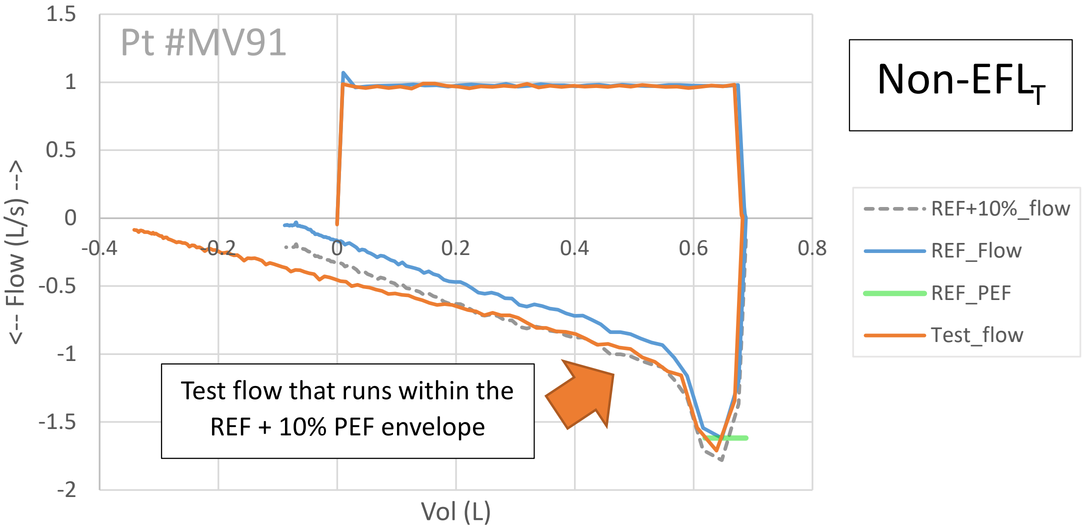

the test flow could be seen running within the reference flow + 10% PEF boundary (example figure on the left). Thus, the parallel of the test curves and the reference curves must also be considered.

The addition of the delta Vte criterion

We determined the “delta Vte” or the difference between the Vte of the test breath and the reference breath during a PEEP reduction test.

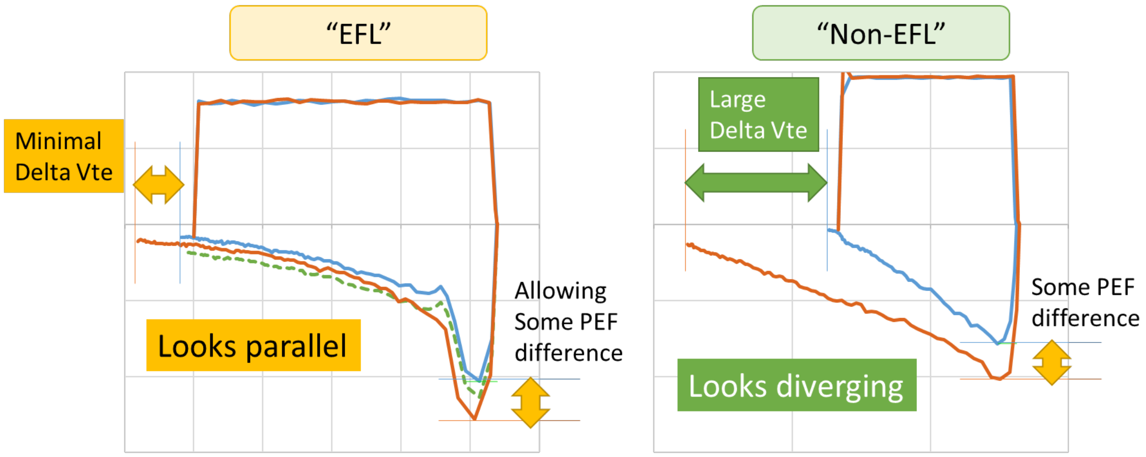

In non-EFL$_T$ case, the Vte in the test breath would increase compared to the reference breath due to its lower end-expiratory lung volume (EELV) being at lower PEEP level. In EFL$_T$, however, due to the trapped air at the upstream segment of the choke point, the EELV is virtually the same as the reference. The Vte of the test breath in EFL$_T$ cases thus minimally increase or being the same as the test breath (“minimal delta Vte”). The cut-off point of this delta Vte has never been reported.

In order to compare the delta Vte in non-EFL VS EFL patients, we have explored our cases with <u>definite non-EFL$_T$</u> (no significant overlap of the curves even with the REF + 10%PEF envelope) VS cases with <u>definite EFL$_T$</u> (significant overlap with the original F-V curve of the reference breath).

$$Percent\ \Delta V_{te} = 100 \times \frac{(TestBreath\,V_{te} - RefBreathV_{te})}{RefBreathV_{te}}$$

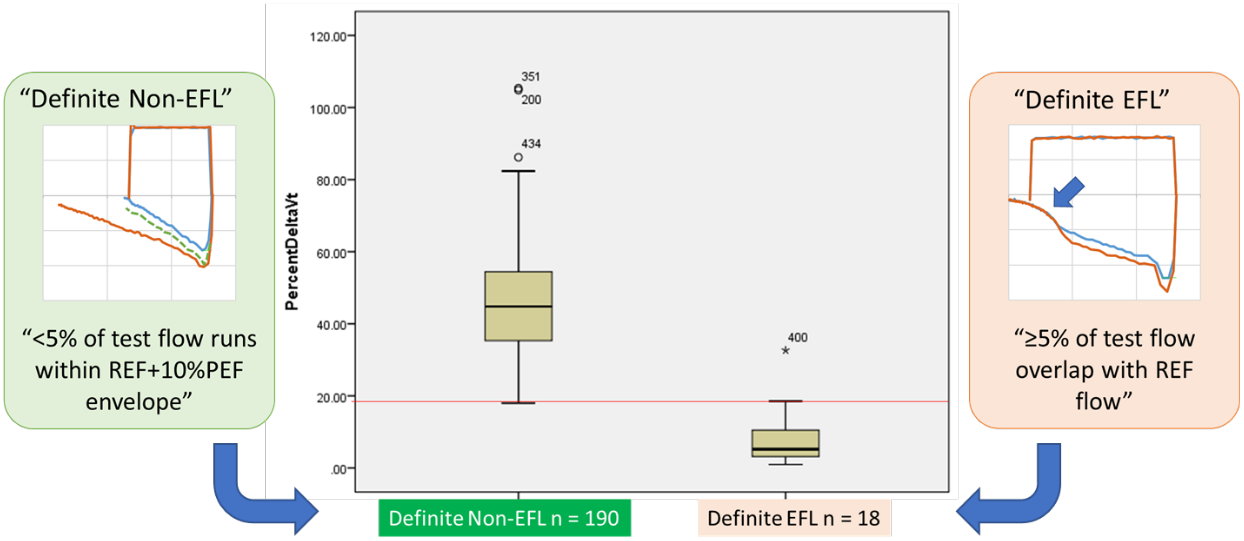

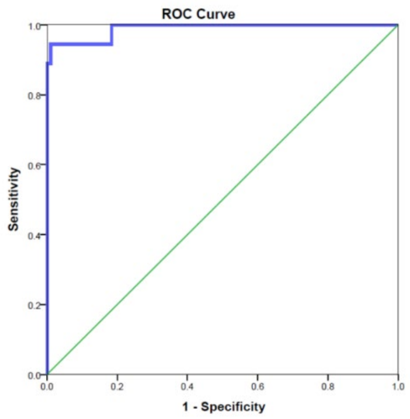

Delta Vte can distinguish between the EFL$_T$ and non-EFL$_T$ in our selected cases ($p < 0.001$) with AUROC 0.989 (95%CI: 0.969-1.000)

We had found the best cut-off point of delta Vte that can distinguish the EFL$_T$ and non-EFL$_T$ cases to be 18.7% of the reference Vte. This was pragmatically rounded to 20%.

The finalized criteria for EFL$_T$ by PEEP reduction manoeuvre

EFL$_T$ at ZEEP is present when the PEEP reduction manoeuvre from 5 cmH_2O to ZEEP produces these results:

(1) the flow-volume curve of the test breath has a significant portion (≥ 5% of the reference Vte) running within the envelope of the reference breath's flow +10% of the reference peak expiratory flow (PEF); and,

(2) the increased exhaled tidal volume during the PEEP reduction (during the test breath) is less than 20% of the reference breath.

End of the ***Section S4: development of EFL$_T$ criteria for the PEEP reduction manoeuvre***

Section S5:
Development of EFL_T criteria for the Rex method

From our hypothesis that, in the case with EFL_T, the instantaneously calculated expiratory airway resistance (Rex_i) should substantially increase to a higher level than the inspiratory resistance (Rin). However, the exact threshold is lacking.

During preliminary analysis, we have observed that, except for the initial part of Rex curve just before the "elbow" point, the Rex curve would form a constant line (approximately) equals to the value of Rin. However, just like the variation of the flow in the PEEP reduction manoeuvre (see section S4), the variation also exists in the Rex measurements. Moreover, based on physiologic grounds, the Rex should probably be higher than the Rin due to the reduction of the airways diamater during expiration. This implies that there should be a margin related to the Rin value to be determined. We have selected 283 non-EFL_T cases (classified by the PEEP reduction manoeuvre from 5 cmH_2O to ZEEP) and analyzed the relationship between the Rex and Rin found in the Rex analysis of these patients at ZEEP.

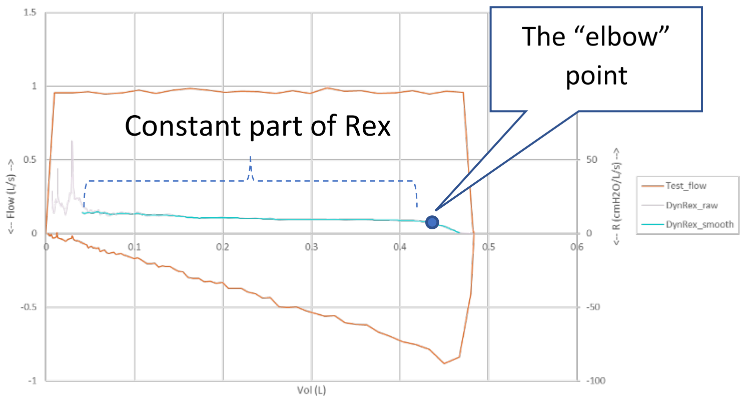

1. Rex analysis from a representative **non-EFL_T case**. This came from the breaths at ZEEP with an end-expiratory pause, up to 3 breaths per case.

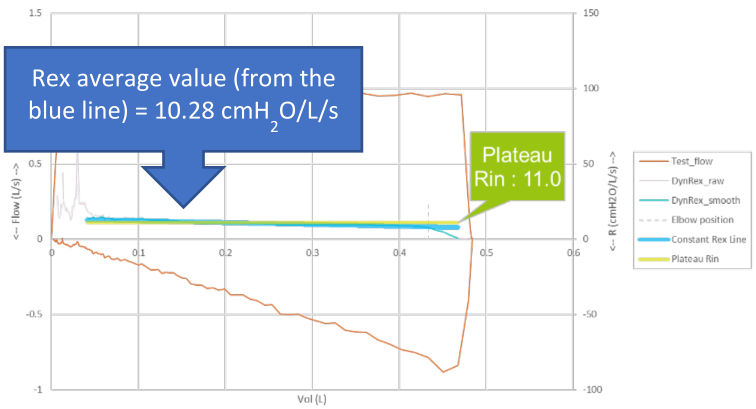

2. In order to estimate the Rex value of the constant portion, we performed a linear fitting equation to the portion after the elbow to yield a near-horizontal straight line. An average value of this straight line is the surrogate value of the Rex.

3. This will then be compared to the "Plateau Rin" of that breath. The Rex and Rin value from all representative breaths of non-EFL_T cases were analyzed using a linear correlation and the Bland-Altman plot.

◆ *Section S5: development of EFL_T criteria for the Rex method*

The relationship between the Rex and Rin in the selected cases

The Rex was analyzable in 281 out of 283 non-EFL_T cases. There were 775 pairs of Rex and Rin available for comparison.

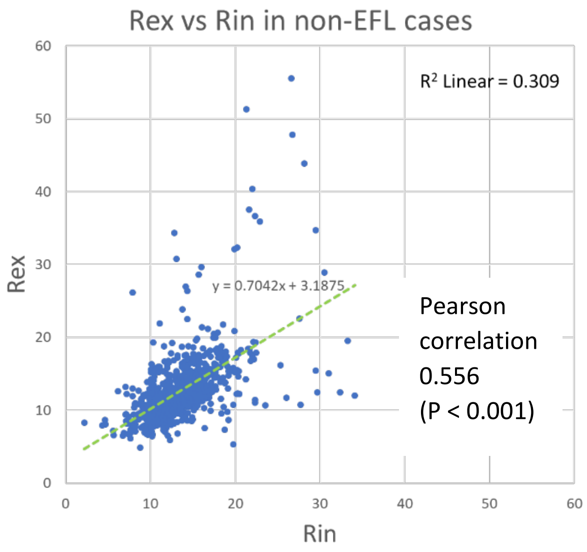

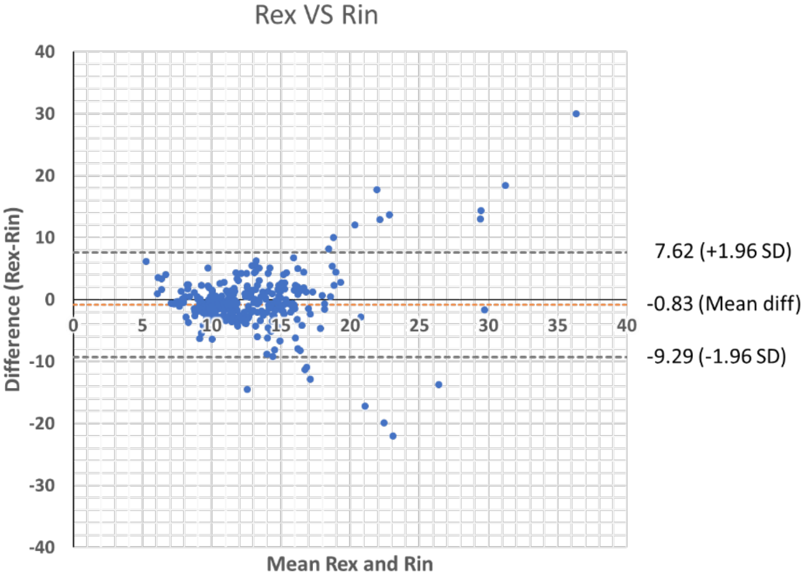

From the Bland-Altman plot, the mean difference of the Rex and Rin is almost equal to zero (-0.83), while the ±1.96 SD range of the difference is +7.62 to -9.29. For pragmatic reason, we had rounded the difference to be ±10 $cmH_2O/L/s$. This converts to the threshold of Rex: “Rex being 10 $cmH_2O/L/s$ or more above the Rin would suggest an EFL_T state”.

In order to be consistent with the EFL_T criteria of the PEEP reduction manoeuvre, the Rex_i has to stay above the threshold for ≥ 5% of Vte to be significant enough to be judged as having EFL_T.

We have finally defined the operational criteria to classify EFL_T by the Rex method as followed.

> EFL_T at a particular PEEP level is defined when the Rex analysis performed at that PEEP level shows:
>
> “A breath where the Rex_i value progressively increases along the expiration, to the value significantly higher (> 10 $cmH_2O/L/s$) than the plateau of its Rin for ≥ 5% of the Vte”

End of the ***Section S5: development of EFL_T criteria for the Rex method***

Section S6: FLOWLY study (validation set for Rex analysis)

The validation set was derived from the data collected in another study: Screening expiratory flow limitation by flow-time curve (FLOWLY). The trial was a single-center, prospective, physiological study conducted at St. Michael's Hospital, Toronto, ON, Canada. Brief details of the study are as follow:

Primary objective:

To test the hypothesis that various parameters derived from flow-time curve can accurately detect the EFL in real time during mechanical ventilation.

Secondary objective:

To test whether the above parameters correlate with the severity of EFL.

Inclusion criteria:

1. Age ≥18 years, intubated or tracheostomized
2. Receiving invasive mechanical ventilation in assist/control mode with PEEP ≥ 5 cmH_2O
3. Receiving continuous intravenous sedation
4. Displaying a monotonous regular breathing pattern with no obvious asynchrony

Exclusion criteria:

1. Severe hypoxemia in baseline clinical ventilator settings (SpO_2 < 90%)
2. Hemodynamic instability (mABP <60 mmHg, SBP >180 mmHg, HR <40/min or >150/min)

Brief study procedures

Once patients are confirmed to meet the inclusion criteria, the patient will be continued to ventilate with their current clinical settings / mode and determine the stability of inspiratory tidal volume (defined by ≤10% variation of the tidal volume between each breath over 5 breaths). PEEP was abruptly reduced by 5 cmH_2O for 3-4 breaths, for two episodes, 5 minutes apart. This allowed diagnosing EFL, using a flow-volume loop during off-line analysis. The end-expiratory and end-inspiratory pause (lasting 2 seconds each) was performed to assess the total PEEP and plateau pressure for the determination of lung mechanics.

Data recording and analysis

The flow and pressure waveforms were collected via Servo ventilators using Servo Tracker v4.1 (Maquet Critical Care AB, Sweden) or from the dedicated pneumotachometer (MP150 systems and AcqKnowledge v4; BIOPAC Systems, Inc., CA, USA) attached to the circuit. The refresh rate was adjusted to 100Hz for Rex analysis using the same software used in the MAFAI VENT study (MAFAI VENT waveform analyzer v 2.74).

Ethical considerations:

The study was conducted in accorded with the principles of the Declaration of Helsinki and with the ICH Guidelines for Good Clinical Practice. The study protocol was submitted and approved by the St. Michael's Hospital's Research Ethics Board before beginning the study (REB# 17-098). The study was registered on clinicaltrials.gov (NCT03215316).

Data set included in this study

Including the cases recruited during Sep 2017 – Jan 2020, we have found 35 analyzable cases to be used as a validation set for the MAFAI VENT study.

Table S1: Baseline ventilator settings, parameters, and lung mechanics at baseline in the overall population, non-EFL_T and EFL_T groups (using PEEP reduction from 5 cmH_2O to ZEEP).

Settings / Parameters	All patients (n = 339)	Non EFL_T group (n = 283)	EFL_T group (n = 56)	P-value
Ventilator model, n (%)				0.287
PB 840	280 (82.6%)	237 (83.7%)	43 (76.8%)	
PB 980	50 (14.7%)	38 (13.4%)	12 (21.4%)	
Hamilton S1	9 (2.7%)	8 (2.8%)	1 (1.8%)	
Tracheostomy tube, n (%)	19 (5.6%)	16 (5.7%)	3 (5.4%)	1
Mode of ventilation at baseline				N/A
Pressure controlled (PCV)	205 (60.5%)	178 (62.9%)	27 (48.2%)	
Pressure support (PSV)	128 (37.8%)	100 (35.3%)	28 (50.0%)	
Volume controlled (VCV)	5 (1.5%)	4 (1.4%)	1 (1.8%)	
Synchronized intermittent mandatory (SIMV)	1 (0.3%)	1 (0.4%)	0 (0)	
Prescribed sedative / analgesic drug(s), n (%) *	124 (36.6%)	102 (36.0%)	22 (39.3%)	0.645
Set PEEP, cmH_2O	5.8 ± 1.3	5.6 ± 1.1	6.3 ± 1.8	0.005
Set Respiratory Rate, min^{-1} †	15.8 ± 3.4	15.7 ± 3.2	15.6 ± 2.8	0.939
Actual Respiratory Rate, min^{-1}	18.6 ± 3.9	18.5 ± 3.8	19.3 ± 4.1	0.153
Actual Mean Vte, mL	479.1 ± 114.8	476.9 ± 113.2	490.1 ± 123.3	0.431
Actual Minute Ventilation, Lpm	8.4 ± 2.5	8.3 ± 2.4	8.8 ± 2.7	0.19
Set FiO_2	0.36 ± 0.10	0.36 ± 0.09	0.35 ± 0.12	0.856
Crs, mL/cmH_2O ‡	43.7 ± 12.2	44.8 ± 11.7	37.9 ± 13.0	<0.001
Airway resistance (inspiration), cmH_2O/L/s ‡	14.8 ± 4.4	14.2 ± 3.8	17.9 ± 5.6	<0.001
Total PEEP, cmH_2O ‡	2.0 (1.5-2.9)	1.8 (1.4-2.4)	5.1 (3.8-7.4)	<0.001

Definition of abbreviations: Crs = Compliance of the respiratory system; FiO_2 = Fraction of inspired oxygen; PEEP = Positive end-expiratory pressure. Categorical variables are described as number (percentage); continuous variables are described as mean ± SD or median (interquartile range), as appropriate.

* Using any of the following drugs at the time of waveform collection: midazolam, propofol, dexmedetomidine, fentanyl, morphine
† Total n = 210 using assist/controlled mode (n = 182 in non EFL_T group and n = 28 in EFL_T group)
‡ Lung mechanics measurements at ZEEP with 2-seconds end-inspiratory and end-expiratory pauses. Data were valid in n = 335 for overall population, n = 279 for non-EFL_T, and n = 56 for EFL_T group

Table S2: The presence of EFL_T of the same patient at ZEEP and at PEEP level ≥ 5 cmH_2O, as determined by Rex analysis. Total n = 440*, and the P-value is < 0.001 (McNemar paired analysis).

		**EFL_T state at PEEP ≥ 5 cmH_2O**		**Total**
		**No EFL_T**	**EFL_T**	
**EFL_T state at ZEEP**	**No EFL_T**	**322** (95.5% of non-EFL_T at ZEEP) (73.2% of total)	**15** (4.5% of non-EFL_T at ZEEP) (3.4% of total)	**337** (76.6%)
	**EFL_T**	**44** (42.7% of EFL_T at ZEEP) (10.0% of total)	**59** (57.3% of EFL_T at ZEEP) (13.4% of total)	**103** (23.4%)
**Total**		**366** (83.2%)	**74** (16.8%)	**440**

When applying PEEP (changing from ZEEP to PEEP ≥ 5 cmH_2O):

- There was 4.5% of cases with NO EFL_T at ZEEP who developed NEW EFL_T
  (This was 3.4% of the total cases)
- There was 42.7% of cases with EFL_T at ZEEP where EFL_T was eliminated when PEEP was applied
  (This was 10.0% of the total cases)

* This included patients with initial set PEEP > 5 cmH_2O which had been excluded in the main analysis

Table S3: The 2 x 2 contingency table for agreements between the Rex method and the 5cmH_2O PEEP reduction method in the validation dataset (FLOWLY study).

	**Rex analysis** **POSITIVE EFL_T**	**Rex analysis** **NEGATIVE EFL_T**	**Total n**
**PEEP reduction** **POSITIVE EFL_T**	7	0	7
**PEEP reduction** **NEGATIVE EFL_T**	3	25	28
**Total n**	10	25	35

Using PEEP reduction from clinical PEEP to clinical PEEP - 5 cmH_2O as a gold standard: The Rex analysis method provides 91.4% agreement (95% CI: 76.9% - 98.2%), 100% sensitivity (95% CI: 59.0% - 100%) and 89.3% specificity (95% CI: 71.8% - 97.7%). The positive and negative predictive value of Rex were 70% (44.5% - 87.2%) and 100% (86.3% - 100%), respectively. The Cohen's k is 0.77 (95% CI: 0.53 – 1.00), i.e., substantial agreement (Landis & Koch, 1977).

In FLOWLY study, the prevalence of EFL_T by PEEP reduction and by Rex method were 20.0% and 28.5%, respectively.

Table S4: Characteristics of patients in the validation set for Rex analysis (FLOWLY study), as classified by the PEEP reduction manoeuvre from clinical PEEP to clinical PEEP – 5 cmH_2O.

Parameters	All patients (n = 35)	Non-EFL_T group (n = 28)	EFL_T group (n = 7)	P-value
Female, n (%)	13 (37.1)	12 (42.9)	1 (14.3)	0.22
Age, years	54.9 ± 13.3	54.7 ± 12.2	55.7 ± 19.0	0.872
Weight, kg*	80.0 ± 22.9	81.9 ± 24.6	72.3 ± 11.8	0.368
Height, cm*	168.7 ± 11.2	168.5 ± 11.7	169.8 ± 7.8	0.842
Body mass index, kg/m^2*	25.2 ± 11.1	25.3 ± 11.9	24.8 ± 3.4	0.939
Body mass index ≥ 30 kg/m^2, n (%)*	7 (22.6)	7 (25.9)	0 (0)	0.55
PaO_2/FiO_2 ratio, median (IQR)	222.0 (134.0 – 282.0)	227.7 (139.8 – 282.5)	173.3 (83.3 – 285.0)	0.514
Main cause of intubation / mech. ventilation, n (%)				
Pulmonary cause	20 (57.1)	15 (53.6)	5 (71.4)	0.672
Hemodynamic cause	4 (11.4)	3 (10.7)	1 (14.3)	1
Neurological cause	4 (11.4)	3 (10.7)	1 (14.3)	1
Post procedure / operation / airway protection	5 (14.3)	5 (17.9)	0 (0)	0.559
Cardiogenic pulmonary edema and volume overload	1 (2.9)	1 (3.6)	0 (0)	1
Other	1 (2.9)	1 (3.6)	0 (0)	1
Underlying disease, n (%) †				
Chronic cardiac disease	6 (17.1)	5 (17.9)	1 (14.3)	1
Chronic lung disease	16 (45.7)	10 (35.7)	6 (85.7)	0.032
COPD	14 (40.0)	10 (35.7)	4 (57.1)	0.401
Asthma	2 (5.7)	1 (3.6)	1 (14.3)	0.365
Cystic fibrosis	2 (5.7)	0 (0)	2 (28.6)	0.035
Lower set PEEP, cmH_2O ‡	3 (0 – 7)	3 (0 – 7)	2 (0 – 4)	0.362
Crs, mL/cmH_2O	47.0 ± 19.9	48.0 ± 21.0	42.9 ± 15.4	0.552
Airway resistance (inspiration), $cmH_2O/L/s$	17.0 ± 5.9	15.1 ± 4.0	24.2 ± 6.9	<0.001
Intrinsic PEEP, cmH_2O §	1.8 (0.9 – 4.1)	1.5 (0.8 – 2.8)	7.5 (6.9 – 8.9)	<0.001

* Data available in n =31 (27 in non-EFL_T group and 4 in EFL_T group)
† A patient could have multiple diseases
‡ The set PEEP level where the lung mechanics, intrinsic PEEP, and EFL_T were determined. This was clinical PEEP – 5 cmH_2O.
§ Intrinsic PEEP = Total PEEP – Set PEEP

Table S5: The distribution of the anatomical site of pathology between each subtype of EFL_T.

**Anatomical site(s) of pathology**	**Early EFL_T n = 27**	**Late EFL_T n = 32**	**Total n = 59**
Airway alone	13 / 27 (48.1%)	1 / 32 (3.1%)	14 / 59 (23.7%)
Non-airway alone (parenchyma or pleura or chest wall)	5 / 27 (18.5%)	25 / 32 (78.1%)	30 / 59 (50.8%)
Both airway & non-airway lesions*	6 / 27 (22.2%)	2 / 32 (6.3%)	8 / 59 (13.6%)
Undetermined †	3 / 27 (11.1%)	4 / 32 (12.5%)	7 / 59 (11.9%)
Total	27	32	59

* e.g. - Acute exacerbation of COPD (airway) due to pneumonia (parenchyma)
- Asthmatic attack (airway) in obese patient (chest wall)

† e.g. A patient without any previous history of airway disease, had no wheezing or rhonchi on the physical examination record, and the CXR or CT chest was clear, without any pleural or chest wall lesion seen

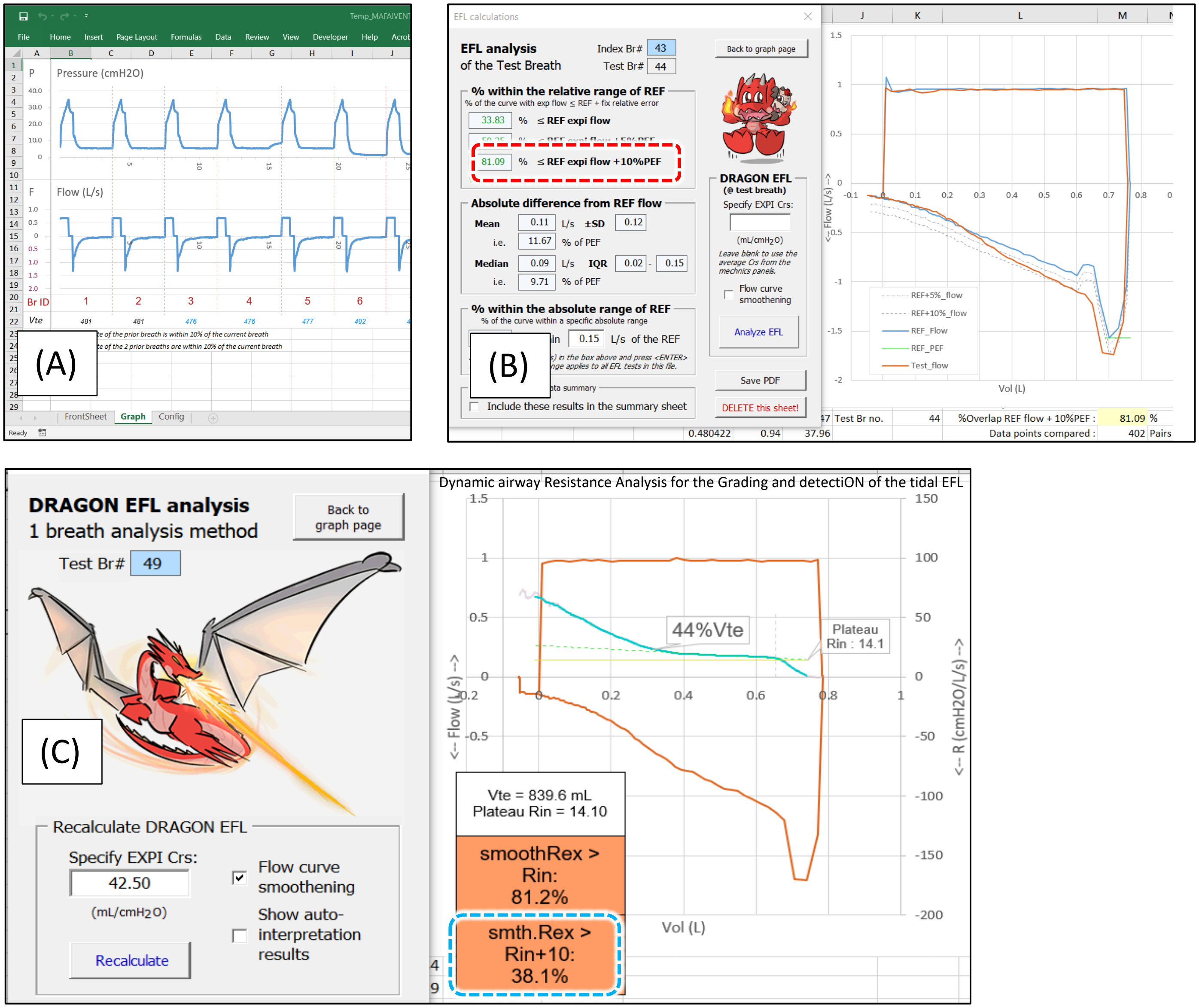

Figure S1: Waveform data analyses in more details. The flow and pressure data from the PB840/980 ventilator (a sampling rate of 50 Hz) were recorded using a serial terminal freeware Termite version 3.4 (Informatie-Technologisch Bureau CompuPhase, Bussum, Netherlands). The data from the Hamilton G5/S1 (31.25 Hz) was recorded by Hamilton Datalogger v5.0 (Hamilton Medical AG, Bonaduz, Switzerland). **Panel A:** The data were imported to MAFAI VENT waveform analyzer v 2.74 (Mahidol University, Bangkok, Thailand). This is a macro-based software developed by the first author (D.J.) and run on Excel 2019 (Microsoft Corp., WA, USA). **Panel B:** Analysis of PEEP reduction manoeuvre, showing the overlapped flow-volume loops of the test breath and the reference breath. The percentage of the overlap was calculated (the red dashed-box). **Panel C:** The Rex method. We transformed the flow and pressure data from time-based value to the volume-based value (one data-point every 2 mL of volume exhaled) using linear extrapolation from the two adjacent data-points. In order to avoid the artifacts at the end of expiration (with very low flow, which will cause the Rex to have extreme fluctuation), we omitted the flow data within the last 5% of the exhaled Vt. With user-specified Crs, the software calculated the Rex value and smoothened the data by 19 data-points moving-average to generate a Rex curve (the blue-green curve). The percentage of Vte that the Rex_i value was higher than the Rin + 10 value was shown (the blue dashed-box).

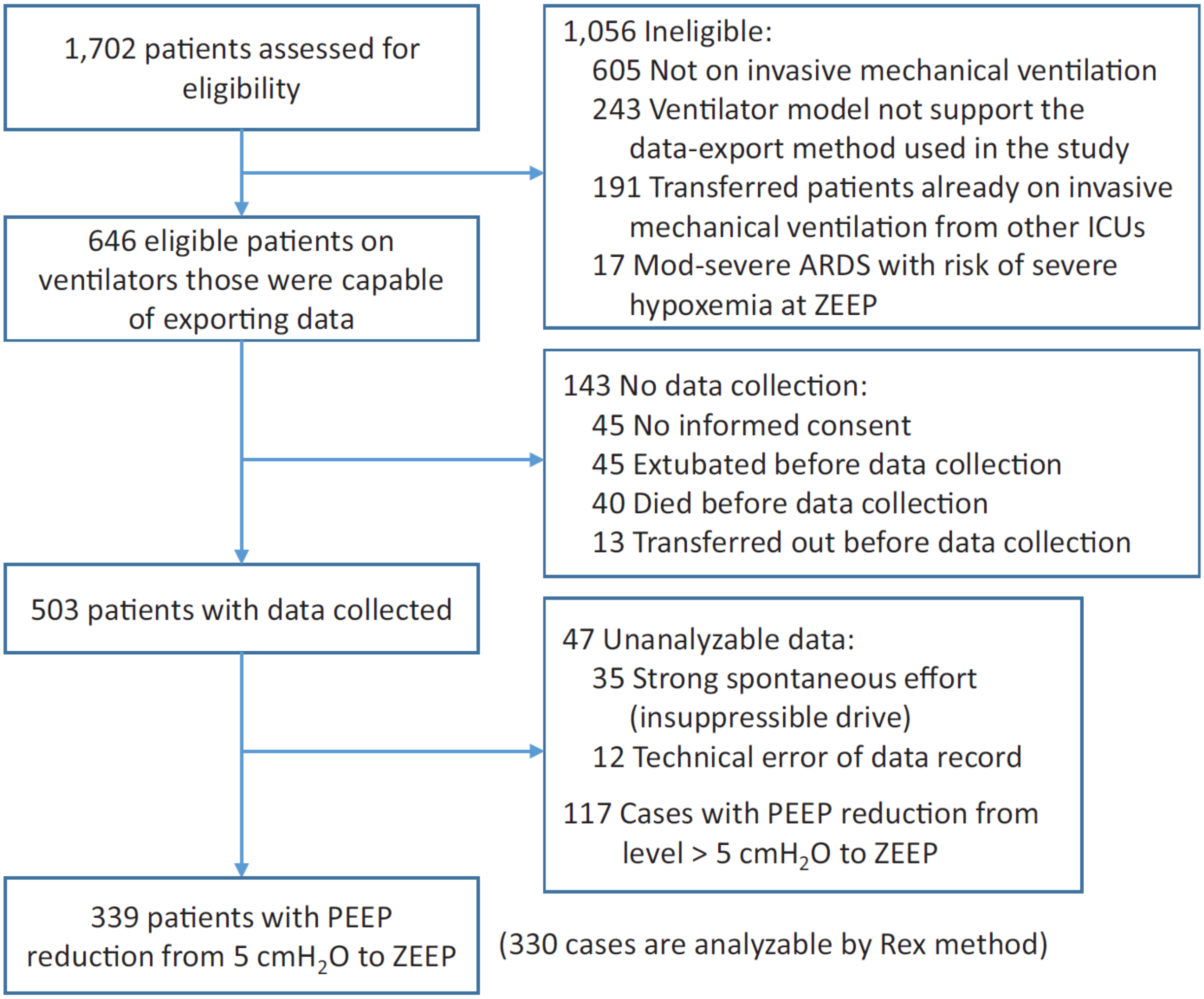

Figure S2: The flow diagram of the patients.

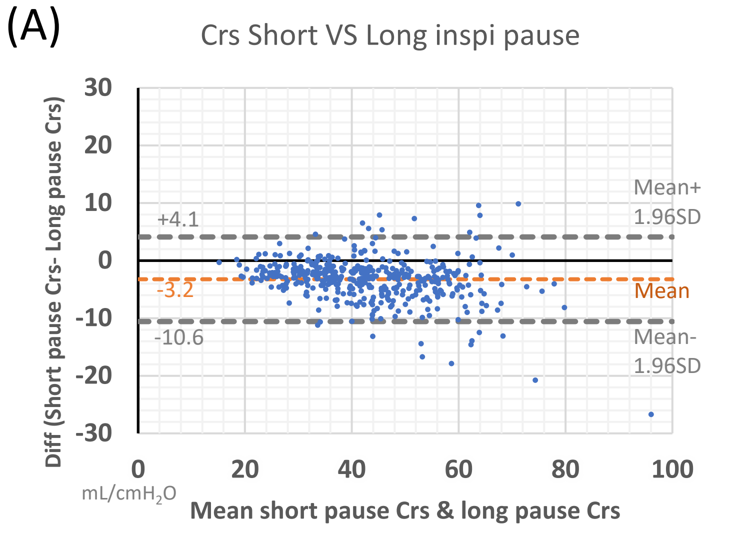

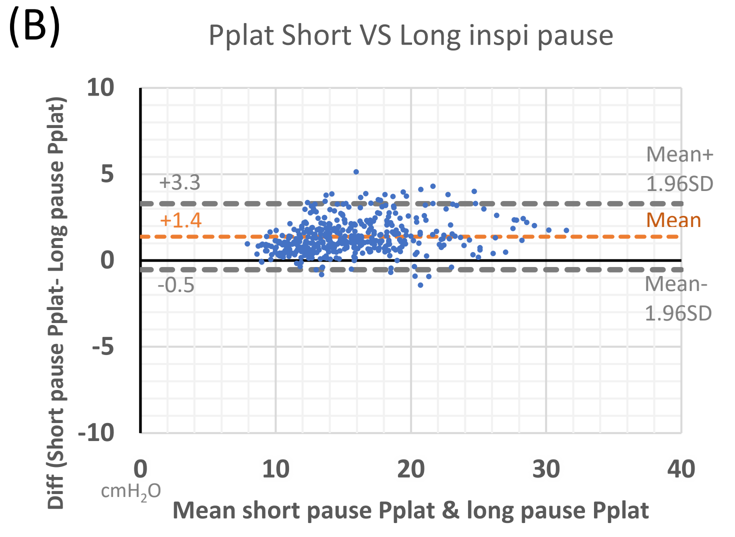

(C) Agreements between the **Rex method using a 2-seconds (long) end-inspiratory pause** and **the standardized PEEP reduction**

	Rex long pause +ve EFL_T	Rex long pause -ve EFL_T	Total n
**PEEP reduction POSITIVE EFL_T**	44	12	56
**PEEP reduction NEGATIVE EFL_T**	21	258	279
**Total n**	65	270	335

Using PEEP reduction from 5 cmH_2O to ZEEP as a gold standard, the Rex analysis (2-seconds end-inspiratory pause) method provides:

Agreement	90.2%	(95% CI: 86.4% - 93.1%)
Sensitivity	78.6%	(95% CI: 65.6% - 88.4%)
Specificity	92.5%	(95% CI: 88.7% - 95.3%)
PPV	67.7%	(95% CI: 57.6% - 76.4%)
NPV	95.6%	(95% CI: 92.9% - 97.3%)
Cohen's k	0.67	(95% CI: 0.56 – 0.77)

i.e., substantial agreement (Landis & Koch, 1977).

(D) Agreements between the **Rex method using a 2-seconds (long) end-inspiratory pause** and **a short (0.2-0.3 seconds) pause**

	Long pause POSITIVE EFL_T	Long pause NEGATIVE EFL_T	Total n
**Short pause POSITIVE EFL_T**	57	9	66
**Short pause NEGATIVE EFL_T**	8	256	264
**Total n**	65	265	330

The Rex analysis by a 2-seconds end-inspiratory pause method and a short-pause method provides:

Agreement	94.9%	(95% CI: 91.9% - 97.0%)
Cohen's k	0.84	(95% CI: 0.76 – 0.91)

i.e., **almost perfect agreement** (Landis & Koch, 1977).

Figure S3: The differences of lung mechanics when using a pragmatic 0.2-0.3 seconds end-inspiratory pause (a short pause) VS a longer 2-seconds pause (a long pause) at ZEEP and the agreements of EFL_T results using these different durations of pause. **Panel A**: The Bland-Altman plots of Crs as measured by a short VS long pause from 431 cases. **Panel B**: The Bland-Altman plots of P_{plat} as measured by a short VS long pause from 431 cases. **Panel C:** Agreements between a long-pause Rex method and the standardized PEEP reduction method in the original dataset (MAFAI VENT). **Panel D:** Agreements between a long-pause Rex method and a short-pause Rex method in the original dataset (MAFAI VENT).

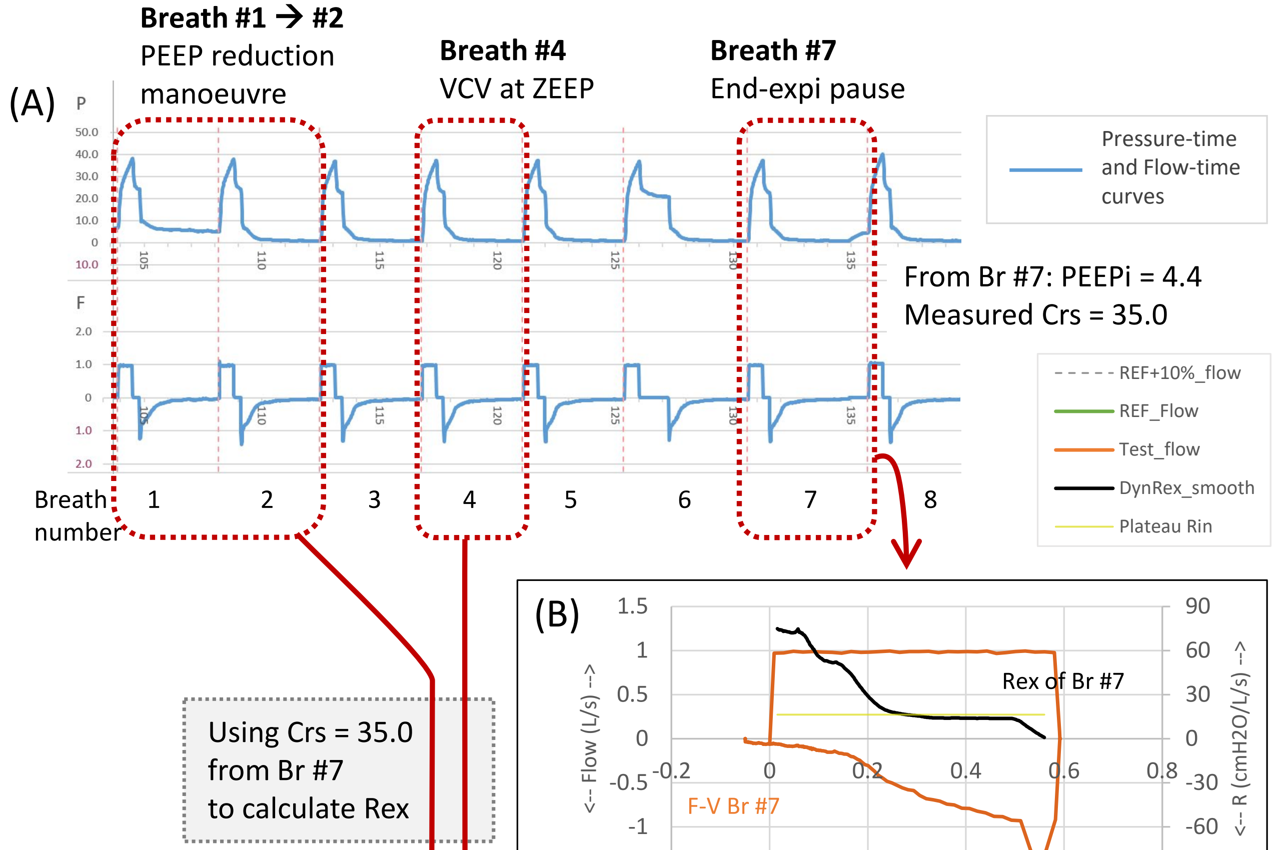

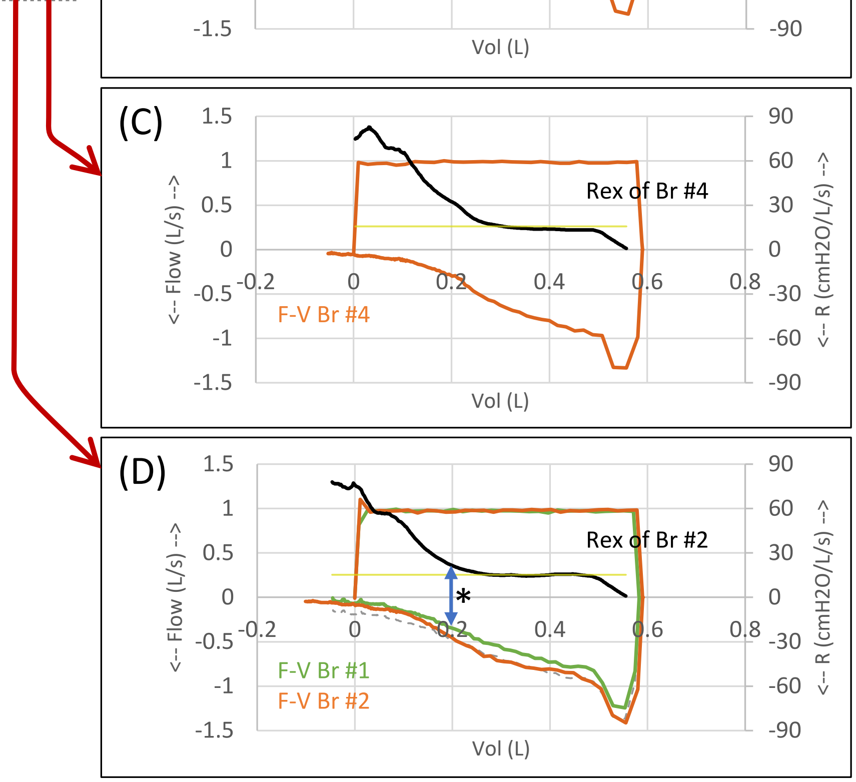

Figure S4: Rex calculations in different breaths with the pre-measured Crs in a patient with EFL_T. **Panel A:** The pressure-time and the flow-time curves using VCV constant flow with end-inspiratory pause of 0.3 seconds. Following a PEEP reduction manoeuvre (PEEP5 in Breath #1 to ZEEP in Br #2), an end-expiratory pause performed in Br #7 revealed PEEPi of 4.4 cmH_2O and Crs = 35.0 mL/cmH_2O. **Panel B:** The Rex calculation using the Crs from the same breath. There is a marked increment of Rex at the end of expiration beyond the plateau of Rin, signifies the presence of EFL_T. **Panel C:** The Rex calculation in another breath (Br #4) using the pre-measured Crs = 35.0 from Br #7. The Rex curve is identical to that of the Br #7 showing the characteristics of EFL_T. **Panel D:** The Rex calculation in the test breath of PEEP reduction manoeuvre (Br #2). This also shows an identical curve of Rex with the characteristics of EFL_T. Moreover, it demonstrates the relationship to the flow change during the PEEP reduction manoeuvre, i.e., the Rex began to rise at the point where the test flow curve started a convergence into the reference flow curve (the two-headed arrow with an asterisk).

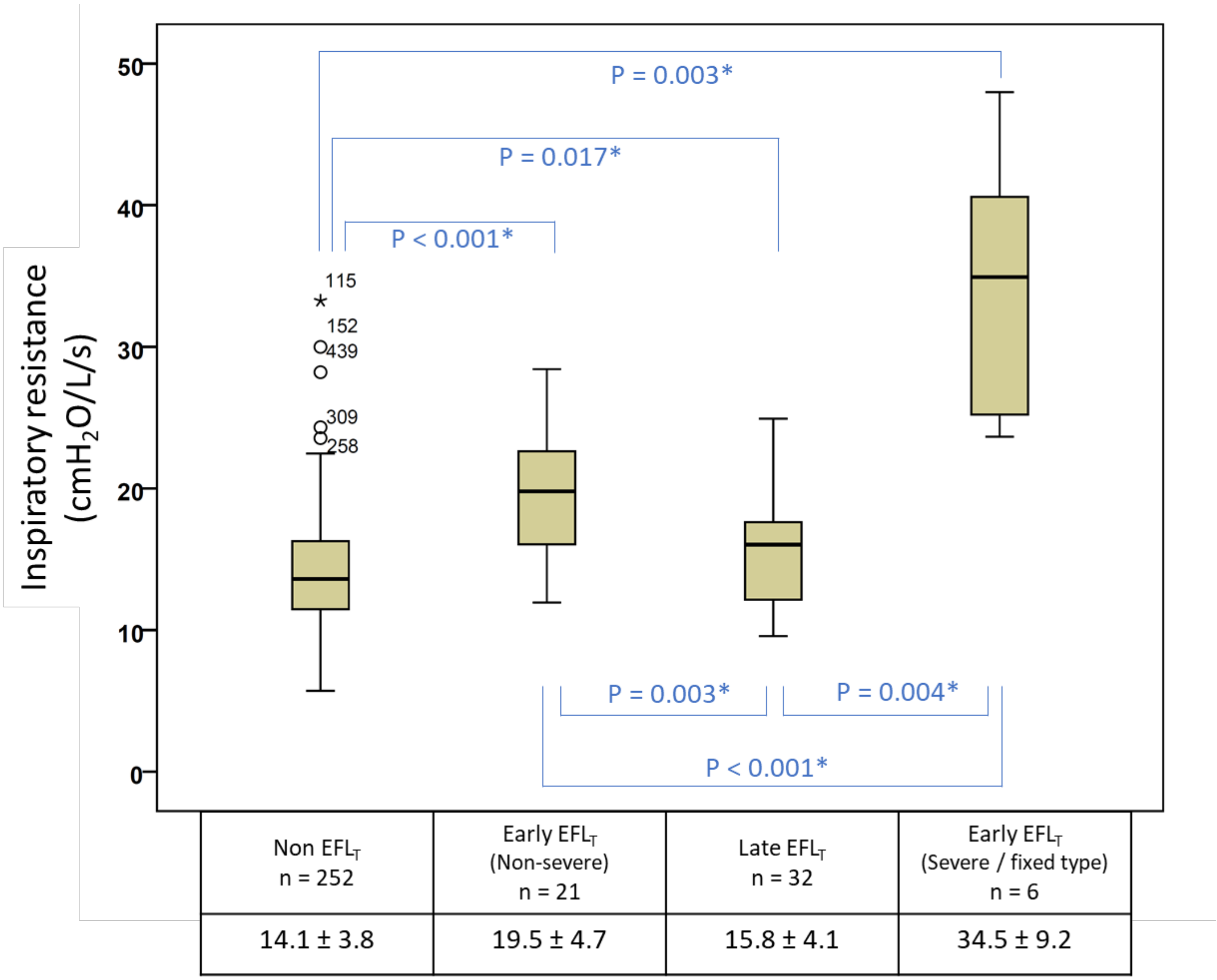

Non EFL_T n = 252	Early EFL_T (Non-severe) n = 21	Late EFL_T n = 32	Early EFL_T (Severe / fixed type) n = 6
14.1 ± 3.8	19.5 ± 4.7	15.8 ± 4.1	34.5 ± 9.2

Figure S5: The differences of inspiratory resistance (Rin), measured with the standard 2-seconds end-inspiratory pause at ZEEP, between non-EFL_T and the proposed subtypes of EFL_T. The early EFL_T (severe / fixed obstruction type) has the highest Rin, followed by the non-severe early EFL_T. Overall comparisons by one-way ANOVA showed statistically significant differences between groups with P-value < 0.001. Asterisks indicate statistically significance by Student's T-test (P < 0.05). Total n = 311 cases.

Section E

Examples of cases and additional materials for discussion

Table E1: The 2 x 2 contingency table for agreements between the simplified "delta Vte only" criterion for the detection of EFL_T, i.e., a delta Vte of less than 20% when performing PEEP reduction from 5 cmH_2O to ZEEP during the VCV mode; VS the "full criteria" considering both the overlapping flow curve and the delta Vte (as used in our primary analysis).

		**Simplified criteria (delta Vte < 20%)**		**Total**
		**No EFL_T**	**EFL_T**	
**Full criteria (flow-volume curve analysis PLUS delta Vte < 20%)** *see main text*	**No EFL_T**	**280** (280/283 = 98.9%)	**3**	**283**
	**EFL_T**	**0**	**56** (56/56 = 100%)	**56**
**Total**		**280**	**59**	**339**

Agreement 99.1% (95% CI: 97.4% - 99.8%), Cohen's K 0.97 (95% CI: 0.93 – 1.00) (almost-perfect agreement, Landis & Koch 1977).

Using "Full criteria" during PEEP reduction from 5 cmH_2O → ZEEP as a gold standard:

- The "delta Vte only" method provides 100% sensitivity (95% CI: 93.6% - 100%) and 98.9% specificity (95% CI: 96.9% - 99.8%).
- The positive and negative predictive value of "delta Vte only" methods are 94.9% (85.8% - 98.3%) and 100% (98.7% - 100%), respectively.

Table E2: Response to external PEEP application among patients with 2 subtypes of EFL_T as determined by the Rex analysis performed at ZEEP.

	**All subtypes of EFL_T at ZEEP n = 103***	**Early EFL_T at ZEEP n = 42**	**Late EFL_T at ZEEP n = 61**	**P-value**
**PEEPi at ZEEP, cmH_2O**	4.16 (2.67 – 6.77)	4.80 (2.68 – 7.50)	4.09 (2.65 – 6.11)	0.226
**Applied PEEP level, cmH_2O †**	5.00 (5.00 – 7.00)	5.00 (5.00 – 6.25)	5.00 (5.00 – 7.00)	0.370
**PEEPi at applied PEEP, cmH_2O ‡**	1.82 (1.34 – 2.73)	2.15 (1.63 – 3.98)	1.66 (1.18 – 2.56)	0.004
**EFL_T was eliminated with PEEP application, n (%) §**	44 (42.7%)	11 (26.2%)	33 (54.1%)	0.005

* This included patients with initial set PEEP > 5 cmH_2O, which had been excluded in the main analysis

† Applied PEEP level was set to 5 cmH_2O or by clinician's initial settings without prior measurement of PEEPi at ZEEP (i.e., the applied PEEP was not systematically set at 80-100% of PEEPi at ZEEP)

‡ Intrinsic PEEP (PEEPi) = Total PEEP – Set PEEP

§ Determined by the Rex analysis result that became non-EFL_T at the applied PEEP level

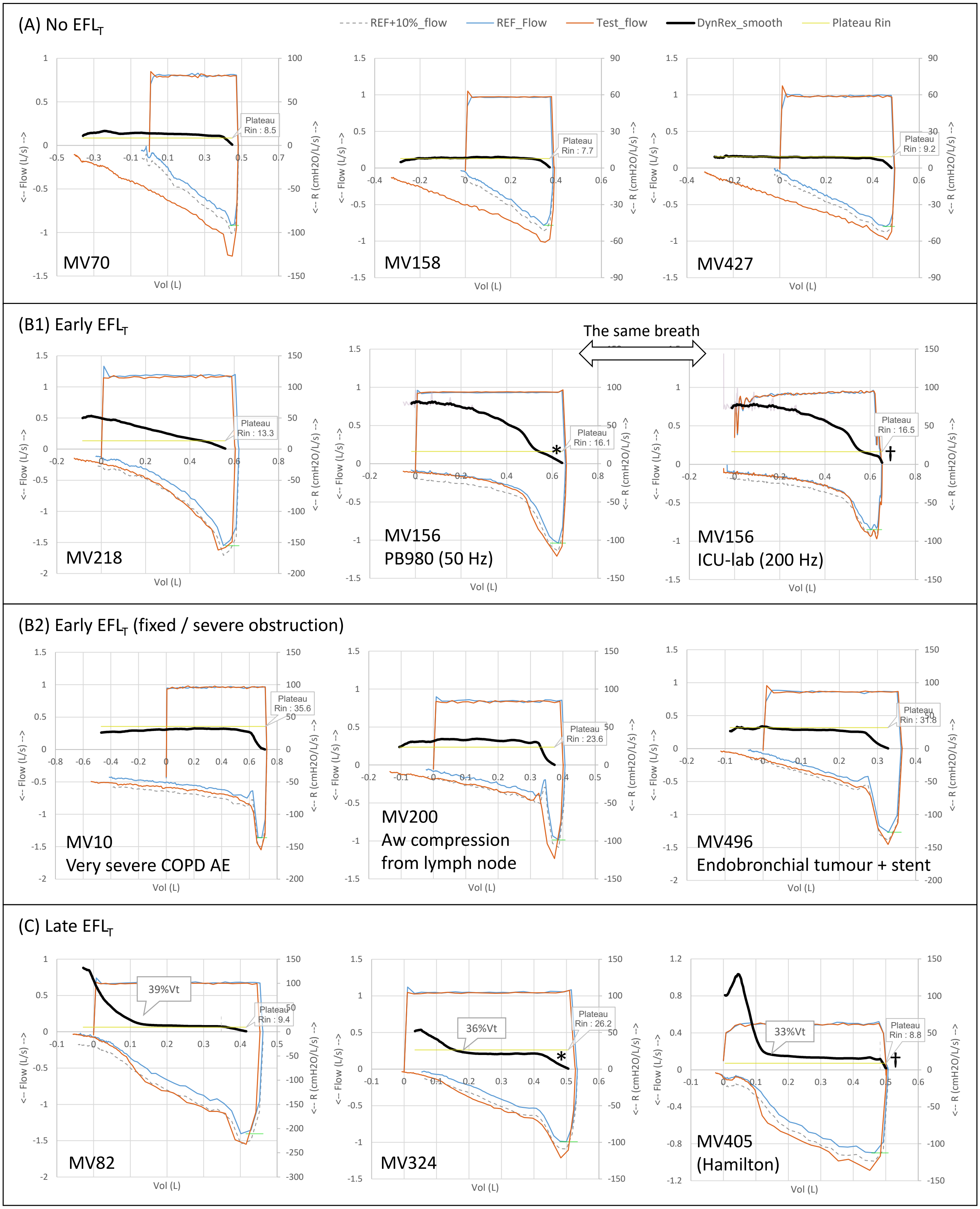

Figure E1: More examples of Rex curves. Data were obtained from PB840/980 ventilators, unless otherwise specified. **Panel A:** Non-EFL_T. **Panel B1:** Early EFL_T. Note the almost-identical Rex curves obtained from a PB980 and a dedicated pneumotachometer (ICU-Lab system; KleisTek Engineering, Bari, Italy). **Panel B2:** Early EFL_T in patients with fixed / very severe obstruction. **Panel C:** Late EFL_T. In #MV324, only the terminal part of the test-breath flow curve showed subtle convergence to the reference-breath curve while the Rex method could clearly detect an EFL_T. This implies a higher sensitivity of the Rex method. **Remark:** Rex curves calculated from the data recorded by PB980 show lower slope at the initial part (asterisks) when compared to the data recorded by sensors those were attached at the wye-piece, e.g., ICU-lab and Hamilton's ventilator (daggers). This probably reflects the phase-shift between the pressure and the flow data due to gas decompression in the circuits.

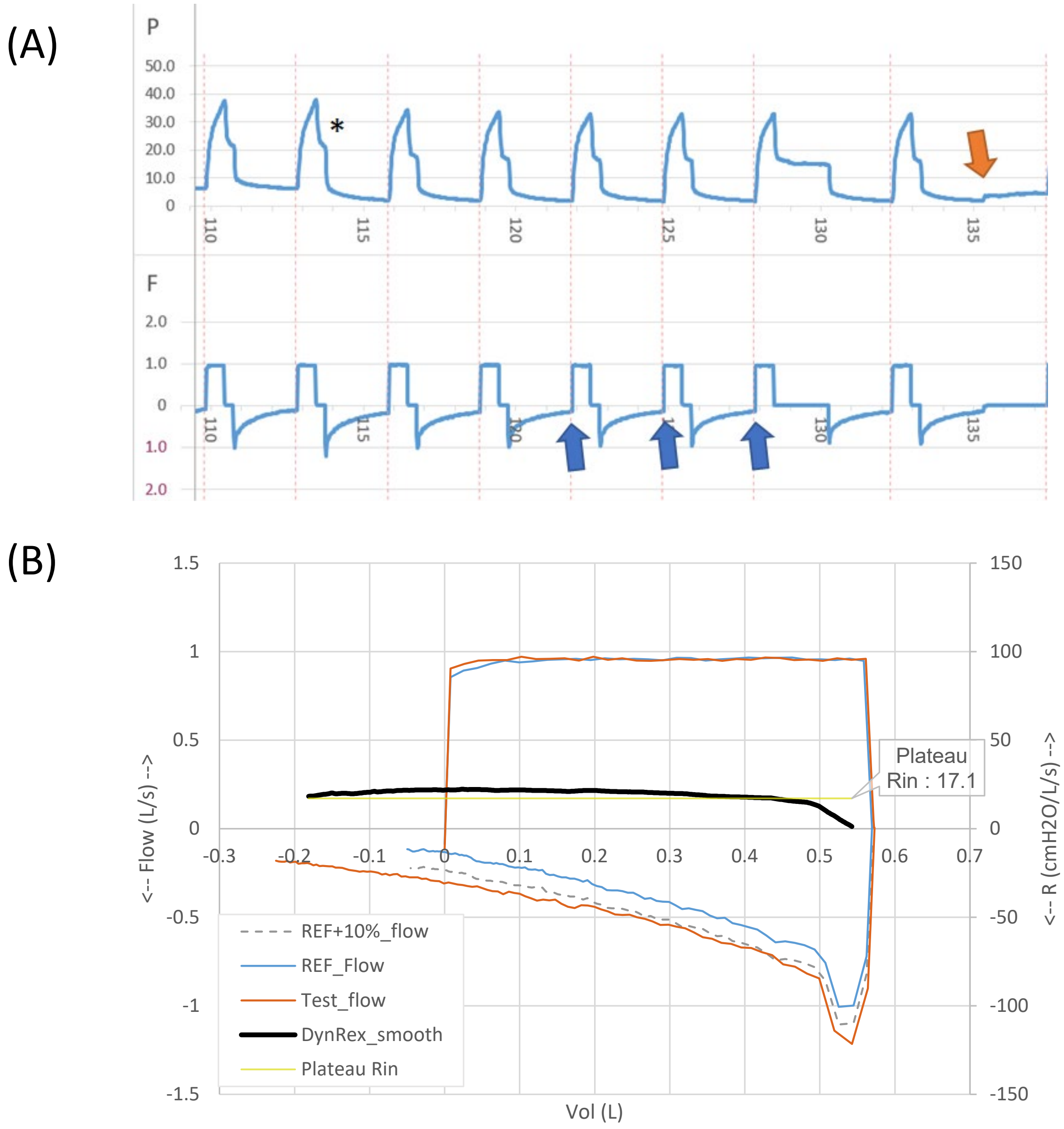

Figure E2: The presence of PEEPi in non-EFL$_T$ cases does not affect the Rex curve. **Panel A:** Pressure-time and flow-time waveforms from patient #MV306 showing an incomplete expiration (the blue arrows), causing PEEPi (4.4 cmH$_2$O at ZEEP, the orange arrow). **Panel B:** The flow-volume curve of the PEEP reduction manoeuvre (from set PEEP = 5 cmH$_2$O to ZEEP, an asterisk in panel A) and the Rex curve from the test breath. The PEEP reduction manoeuvre shows no significant overlap of the test-breath flow on the threshold envelope, indicating no EFL$_T$. The Rex curve also shows a constant, non-EFL$_T$ pattern. Thus PEEPi, in this case, was caused solely by a high respiratory rate and a relatively short expiratory time, not by EFL$_T$.

References for supplemental documents

E1. Pankow W, Podszus T, Gutheil T, Penzel T, Peter J, Von Wichert P. Expiratory flow limitation and intrinsic positive end-expiratory pressure in obesity. J Appl Physiol (1985). 1998;85:1236-1243.

E2. Ferretti A, Giampiccolo P, Cavalli A, Milic-Emili J, Tantucci C. Expiratory flow limitation and orthopnea in massively obese subjects. Chest. 2001;119:1401-1408.

E3. Koutsoukou A, Koulouris N, Bekos B, Sotiropoulou C, Kosmas E, Papadima K, et al. Expiratory flow limitation in morbidly obese postoperative mechanically ventilated patients. Acta Anaesthesiol Scand. 2004;48:1080-1088.

E4. Junhasavasdikul D, Telias I, Grieco DL, Chen L, Gutierrez CM, Piraino T, et al. Expiratory Flow Limitation During Mechanical Ventilation. Chest. 2018;154:948-962.

E5. Volta CA, Dalla Corte F, Ragazzi R, Marangoni E, Fogagnolo A, Scaramuzzo G, et al. Expiratory flow limitation in intensive care: prevalence and risk factors. Crit Care. 2019;23:395.

E6. Hulley SB, Cummings SR, Browner WS, Grady D, Newman TB: Designing Clinical Research, 4th edn: Wolters Kluwer/Lippincott Williams & Wilkins; 2013. Appendix 6B, page 75.

E7. Fleiss JL, Tytun A, Ury HK. A simple approximation for calculating sample sizes for comparing independent proportions. Biometrics. 1980;36:343-346.

E8. Spadaro S, Caramori G, Rizzuto C, Mojoli F, Zani G, Ragazzi R, et al. Expiratory Flow Limitation as a Risk Factor for Pulmonary Complications After Major Abdominal Surgery. Anesth Analg. 2017;124:524-530.

E9. Fogagnolo A, Spadaro S, Karbing DS, Scaramuzzo G, Mari M, Guirrini S, et al. Effect of expiratory flow limitation on ventilation/perfusion mismatch and perioperative lung function during pneumoperitoneum and Trendelenburg position. Minerva Anestesiol. 2023.

E10. Kondili E, Alexopoulou C, Prinianakis G, Xirouchaki N, Georgopoulos D. Pattern of lung emptying and expiratory resistance in mechanically ventilated patients with chronic obstructive pulmonary disease. Intensive Care Med. 2004;30:1311-1318.

E11. Natalini G, Tuzzo D, Rosano A, Testa M, Grazioli M, Pennestri V, et al. Effect of external PEEP in patients under controlled mechanical ventilation with an auto-PEEP of 5 cmH2O or higher. Ann Intensive Care. 2016;6:53.

E12. Marangoni E, Alvisi V, Ragazzi R, Mojoli F, Alvisi R, Caramori G, et al. Respiratory mechanics at different PEEP level during general anesthesia in the elderly: a pilot study. Minerva Anestesiol. 2012;78:1205-1214.

E13. Jones MH, Davis SD, Kisling JA, Howard JM, Castile R, Tepper RS. Flow limitation in infants assessed by negative expiratory pressure. Am J Respir Crit Care Med. 2000;161:713-717.

E14. Lourens MS, Berg BV, Hoogsteden HC, Bogaard JM. Detection of flow limitation in mechanically ventilated patients. Intensive Care Med. 2001;27:1312-1320.

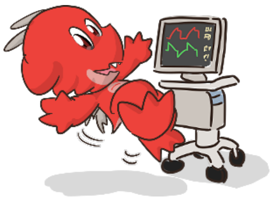

The MAFAI VENT investigators

Chayanon Songsomboon<br>Detajin Junhasavasdikul<br>Namsai Pukiat<br>Tanakorn Tassaneyasin<br>Tananchai Petnak<br>Yuda Sutherasan	- Division of Pulmonary and Pulmonary Critical Care, Department of Medicine, Faculty of Medicine Ramathibodi Hospital, Mahidol University, Bangkok, Thailand.
Akarawut Kasemchaiyanun<br>Kridsanai Gulapa	- Division of Pulmonary and Pulmonary Critical Care, Department of Medicine, Faculty of Medicine Ramathibodi Hospital, Mahidol University, Bangkok, Thailand.<br>- Division of Critical Care, Department of Medicine, Faculty of Medicine Ramathibodi Hospital, Mahidol University, Bangkok, Thailand.
Pongdhep Theerawit	- Division of Critical Care, Department of Medicine, Faculty of Medicine Ramathibodi Hospital, Mahidol University, Bangkok, Thailand.
Sarawut Panichaporn	- Department of Medicine, Faculty of Medicine Ramathibodi Hospital, Mahidol University, Bangkok, Thailand.
Pawin Numthavaj<br>Ratchainant Thammasudjarit<br>Yang Junwei	- Section of Clinical Epidemiology and Biostatistics, Faculty of Medicine Ramathibodi Hospital, Mahidol University, Bangkok, Thailand.
Chanon Puttanawarut	- Chakri Naruebodindra Medical Institute, Faculty of Medicine Ramathibodi Hospital, Bangkok Thailand
